# GenOtoScope: Towards automating ACMG classification of variants associated with congenital hearing loss

**DOI:** 10.1371/journal.pcbi.1009785

**Published:** 2022-09-21

**Authors:** Damianos P. Melidis, Christian Landgraf, Gunnar Schmidt, Anja Schöner-Heinisch, Sandra von Hardenberg, Anke Lesinski-Schiedat, Wolfgang Nejdl, Bernd Auber

**Affiliations:** 1 L3S Research Center, Leibniz University Hannover, Hannover, Germany; 2 Department of Human Genetics, Hannover Medical School, Hannover, Germany; 3 Department of Otorhinolaryngology, Hannover Medical School, Hannover, Germany; 4 Hearing4all Cluster of Excellence, Hannover Medical School, Hannover, Germany; 5 Knowledge-based Systems Laboratory, Leibniz University Hannover, Hannover, Germany; Hebrew University of Jerusalem, ISRAEL

## Abstract

Since next-generation sequencing (NGS) has become widely available, large gene panels containing up to several hundred genes can be sequenced cost-efficiently. However, the interpretation of the often large numbers of sequence variants detected when using NGS is laborious, prone to errors and is often difficult to compare across laboratories. To overcome this challenge, the American College of Medical Genetics and Genomics and the Association for Molecular Pathology (ACMG/AMP) have introduced standards and guidelines for the interpretation of sequencing variants. Additionally, disease-specific refinements have been developed that include accurate thresholds for many criteria, enabling highly automated processing. This is of particular interest for common but heterogeneous disorders such as hearing impairment. With more than 200 genes associated with hearing disorders, the manual inspection of possible causative variants is particularly difficult and time-consuming.

To this end, we developed the open-source bioinformatics tool GenOtoScope, which automates the analysis of all ACMG/AMP criteria that can be assessed without further individual patient information or human curator investigation, including the refined loss of function criterion (“PVS1”). Two types of interfaces are provided: (i) a command line application to classify sequence variants in batches for a set of patients and (ii) a user-friendly website to classify single variants.

We compared the performance of our tool with two other variant classification tools using two hearing loss data sets, which were manually annotated either by the ClinGen Hearing Loss Gene Curation Expert Panel or the diagnostics unit of our human genetics department. GenOtoScope achieved the best average accuracy and precision for both data sets. Compared to the second-best tool, GenOtoScope improved the accuracy metric by 25.75% and 4.57% and precision metric by 52.11% and 12.13% on the two data sets, respectively. The web interface is accessible via: http://genotoscope.mh-hannover.de:5000 and the command line interface via: https://github.com/damianosmel/GenOtoScope.

## Author summary

New high-throughput sequencing technologies can produce massive amounts of information and are used by laboratories to explain the often complex genetic etiology of hereditary diseases. To use these sequencing technologies effectively, software tools have been developed that can aid researchers interpreting genetic data by semi-automatically classifying the biologic (and, thus, potentially medical) impact of the detected variants (i.e. alterations of the patient’s genome compared to the human reference genome). Variant classification itself is a very complex process: various information has to be taken into account and carefully judged, allowing room for interpretation. Over the last decade, the quality of genetic analysis evaluation and the consistency between different laboratories have improved substantially. This is in particular a result of increasing standardization, which extends to gene- or disease-specific considerations. Hereby, a largely uniform variant interpretation is possible even for common but heterogeneous disorders.

To design a reliable tool that can accommodate these precise specifications, we chose hearing loss as a model disease because it is the most common sensory disorder, often hereditary and has a high impact on patients every-day lifes. The currently available genetic variant classification tools are either not designed specifically for the interpretation of variants detected in subjects with hearing loss or they do not allow researchers to use them for batch classification of all variants detected, e.g. in a study group. To address this drawback, we developed GenOtoScope, an open-source tool that automates the pathogenicity classification of variants potentially associated with congenital hearing loss. By adjusting specific parameters, the algorithm could also be easily adapted to other medical conditions. GenOtoScope can be applied for the automatic classification of all variants detected in a set of individuals.

This is a *PLOS Computational Biology* Software paper.

## Introduction

Due to the establishment of modern high-throughput next generation sequencing (NGS) technologies, an ever-increasing amount of sequencing data can be generated. Nevertheless, a whole exome sequencing (WES) file contains approximately 60,000 variants per individual. Consequently, laboratories have to overcome the hurdle of processing this vast amount of data to link the genotype to the phenotype [1]. Notably, the manual classification of variants, by expert curators, is not only time-consuming, but even more, prone to inconsistent functional interpretation and pathogenicity classification of a variant between distinct laboratories [2].

**Fig 1:**
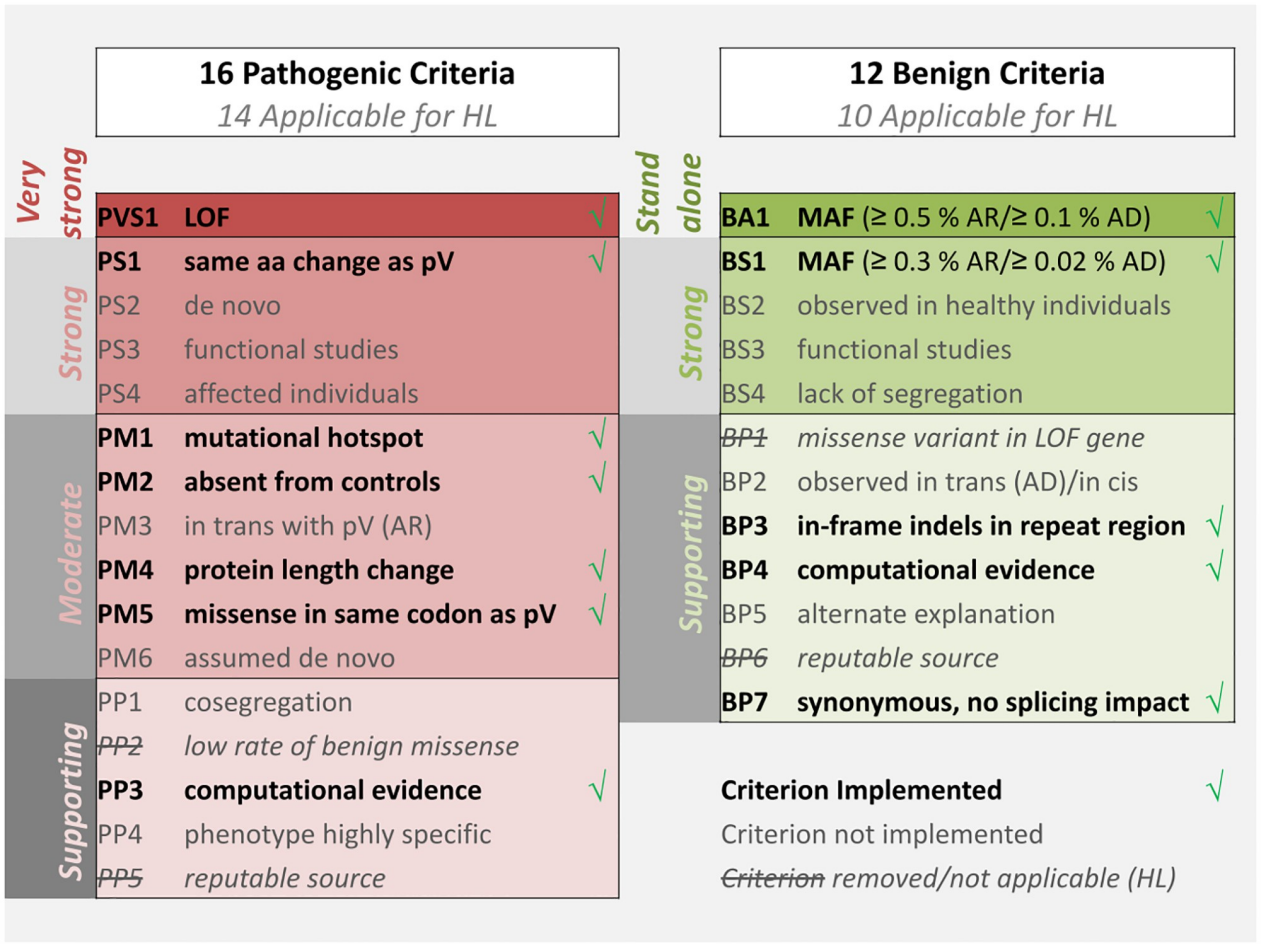
Overview of ACMG/AMP evidence-based criteria. Green marks show implemented criteria. Grey font shows not implemented criteria. Striked-through shows removed or not applicable criterion for hearing loss. Thresholds shown for BA1 and BS1 are specific for HL.

To address this challenge, the American College of Medical Genetics and the Association for Molecular Pathology (ACMG/AMP) have published a set of evidence-based criteria to classify patient variants in five classes of pathogenicity, “benign” (class 1), “likely benign” (class 2), “variants of uncertain significance” (“VUS”) (class 3), “likely pathogenic” (class 4), and “pathogenic” (class 5) [3]. According to these guidelines, various information about a variant of interest and its associated phenotype (e.g., population data, computational data, functional data, segregation data) can be assorted into 28 well-defined categories that function as evidence criteria for a variant to be pathogenic or benign. The acronym of each criterion is a composite of P (pathogenic) or B (benign) and the respective graded strength level, A (stand-alone), VS (very strong), S (strong), M (moderate), P (supporting), followed by a numerical identifier denoting different types of information. The graded combination of evidence criteria results in the five-tier classification system mentioned above. An overview of the evidence-based criteria is depicted in Fig 1 and the classification scheme is shown in S1 Fig.

To specialize for a diverse set of phenotypes with distinct penetrance, allelic and genetic heterogeneity, ACMG has updated its classification criteria for specific hereditary diseases, for example hereditary (breast/ovarian) cancer [4] or cardiomyopathy [5], through the ClinGen Variant Curation Expert Panels (VCEP). Even more than the general ACMG recommendations, these disease-specific criteria are predestined for a computerized approach due to their precise thresholds.

Hearing loss (HL) is the most common sensory disorder with a high impact on the quality of social and work life of the patient. A genetic etiology can be linked to approximately 50% of the affected individuals [6]. Besides various forms of nonsyndromic hearing loss (NSHL) affecting only the function of the ear, HL can also be a symptom of a superordinate disorder involving other organ systems (syndromic hearing loss). Thus, HL is very heterogeneous with well over 100 genes known to be associated with monogenetic NSHL and more than 400 distinctive syndromes including HL as one of their characteristic symptoms as well [6]. Because of this tremendous versatility combined with the high overall frequency of occurrence, we selected HL as a model disease for the development of our software tool. Therefore, the algorithms presented in this work are by default set to HL-specific thresholds, but can be easily modified to suit other medical conditions.

There are orthogonal approaches to perform the challenging classification of HL variants. The first approach is to use machine learning models that predict the pathogenicity of variants with respect to hearing loss phenotype such as DVPred [7]. However, such approaches do not output the triggered ACMG evidence-based criteria supporting the classification result, thus the classification results cannot be easily interpreted by the human variant curator. An alternative is to gather and organize all known and classified variants for HL into an open-access database. Gene4HL [8] is a database where the human curator can import, export and find a HL variant of interest. Nevertheless, the database approach has limited validity in the variant assessment process, assuming that the human curator aims to assess the pathogenicity of a variant that is not already catalogued in the database. The last approach is to automate ACMG/AMP guidelines specified for HL. In this work, we focus on such an approach. The main reasons are that such a bioinformatics tool will annotate and classify all variants of WES experiment with respect to HL phenotype and output the triggered ACMG evidence-based criteria supporting each pathogenicity classification. Consequently, this tool provides to the human curator, pathogenicity classification for a variant not yet classified and reports the triggered ACMG/AMP criteria allowing for an interpretable classification.

[9] work has introduced disease-specific evidence-based ACMG/AMP criteria, to facilitate the challenging classification of variants for HL. Noteworthy, 2 criteria were marked as not applicable (PP2 and BP1) for HL and 2 criteria (PP5 and BP6) have been sorted out as generally not applicable. Application of the remaining 24 adjusted criteria has been shown to achieve better classification performance compared to the standard evidence-based criteria for known HL-related variants [10]. In the following, we will refer to ClinGen Hearing Loss Gene Curation Expert Panel commitee as VCEP-HL.

A recently published bioinformatics tool, VIP-HL [11], automates 13 out of the 24 evidence-based criteria specified for HL. However, VIP-HL is an online tool that accepts only a single variant per time, thus hindering the automatic and time-efficient interpretation of all variants of WES files for a set of investigated patients, for a heterogeneous condition as HL. To address this limitation of VIP-HL, we present GenOtoScope, a bioinformatics tool, which computes the pathogenicity class and pathogenicity probability for each variant of the input genomic variant file (VCF), based on [9] and [12]. To this end, we designed and implemented algorithms to automate all the evidence-based criteria that need no further individual patient information or human curator investigation. This has resulted in 12 implemented criteria, out of the 24 criteria in total, namely PVS1 (all strengths), PS1, PM1, PM2 (PM2 supporting), PM4, PM5 (PM5 strong), PP3, BA1, BS1 (BS1 supporting), BP3, BP4 and BP7. We provide GenOtoScope as an open-source project, accessible as command line application to classify the WES patient files and as an online tool to classify a single genomic variant of interest.

We benchmarked the performance of GenOtoScope compared to two established classification tools, InterVar [13] and VIP-HL, in two HL data sets. These data sets consist of manually curated HL variants. GenOtoScope outperformed the other two classification algorithms, both, in terms of accuracy and precision. Finally, we investigated the reasons for this superior performance of GenOtoScopeby calculating the difference between the activation frequencies of a tool over the manual curation, for each evidence-based criterion.

In summary, our contributions are:

- introduce GenOtoScope in two forms, a command line application for bioinformatics experts to classify WES VCF files of a set of patients and a web-based application for non-bioinformatics experts to classify single variants.
- compare GenOtoScope classification performance to InterVar and VIP-HL for two manually annotated HL data sets.
- make GenOtoScope an open-source bioinformatics tool, therefore enabling the research community to extend the tool for other diseases.

## Materials and methods

### Automating the examination of ACMG evidence-based criteria

GenOtoScope implements 12 out of 24 ACMG evidence-based criteria specified for hearing loss [9]. More specifically these criteria are the PVS1 (all strengths), PS1, PM1, PM2 (PM2 supporting), PM4, PM5 (PM5 strong), PP3, BA1, BS1 (BS1 supporting), BP3, BP4 and BP7. Based on class category the implemented criteria are sorted in seven pathogenic and five benign criteria. With respect to the data types needed for ACMG criteria, we categorized our implemented criteria into three population data criteria, eight computational and predictive data criteria and one functional data criterion. The comparison of GenOtoScope with VIP-HL and InterVar is summarized in Table 1.

**Table 1:** Overview of ACMG classification tools benchmarked against GenOtoScope.

Tool	Implemented Criteria	Phenotype-specific	Open Code Implementation	Open Annotation Data Sets	Command-line Application (variant sets)	Web Application (single variant)	Evaluation Data Sets
InterVar	18/28 Benign: 8/12 Pathogenic: 10/16	No	Yes	No	Yes	Yes	*De novo* variants in neurodevelopmental disorders (9,305)* Benign & pathogenic ClinVar (49,167)* Pathogenic HGMD (616)* All CLINVITAE (11,696)*
VIP-HL	13/24 Benign: 6/10 Pathogenic: 7/14	Yes	No	Yes	No	Yes	Pilot VCEP-HL (50)** All deafness-related ClinVar (4,948)*
GenOtoScope	12/24 Benign: 5/10 Pathogenic: 7/14	Yes	Yes	Yes	Yes	Yes	All VCEP-HL (158)** Manually classified by diagnostics unit of MHH (118)**

* Classification not based on ACMG/AMP.

** Classification based on ACMG/AMP guidelines specified for HL, by manual curators. (): Number of applicable variants.

The unimplemented criteria by GenOtoScope are 12. These criteria are: PS2, PS3, PS4, PM3, PM6, PP1, PP4, BS2, BS3, BS4, BP2 and BP5. The main reasons not to implement these criteria are: (i) the lack of established processing algorithm (ii) the lack of data and (ii) further patient information. That is, for the criteria needing functional data, PS3 and BS3, there are no established algorithms that could automatically extract the result of a functional study publication for a given human variant. Concerning the lack of data, the examination of the PS4 criterion could not be automated, because there is no database to contain the prevalence of affected and control individuals for all possible variant types. Equally, there is no database with the respective information to automate BS2 and BP2 criteria. Last, the need for genomic data of the patient’s family disables the examination of the segregation data criteria: PS2, PM3, PM6, PP1, PP4, BS4 and BP5.

The number of missing implemented criteria is competitive with the other classifications tools. VIP-HL implements only one extra criterion, BS2. The main reason not to also implement BS2 is that VIP-HL uses particular thresholds, which are not specified by ACMG HL original work, and thus it may not reflected all penetrance and inheritance modes of all HL-related genes.

InterVar implements 18 out of the 28 ACMG original criteria [3]. As explained in the introduction, disease-specific ACMG criteria may vary from the original 24 criteria. Therefore the PP2, PP5, BP1 and BP6 criteria automated by InterVar are not applicable for HL. The remaining two criteria automated by InterVar and not by GenOtoScope are PS4 and BS2. To automate these criteria, InterVar uses the ANNOVAR annotation tool [14]. However, this tool implements PS4 using a general threshold on a phenotype-based GWAS catalog, consequently the called enriched pathogenic variants may not include all HL-relevant variants. Similarly, to automate BS2 criterion, InterVar uses the zygotic information of a healthy individual in the 1000 Genomes project [15] based on the inheritance mode of the variant. Nevertheless, specific thresholds of healthy individuals should be used for HL, which are not published by [9]. As a consequence, there may exist false negative cases; InterVar should activate PS4 or BS2 for a given HL variant but it does not. Finally, the remaining criteria need manual curation or additional information not publicly available (e.g. segregation or phenotypic data), therefore they are not implemented by any of the three classification tools.

In our thorough evaluation, shown in the results section, we demonstrate that regarding the 12 ACMG criteria processed by all three tools, GenOtoScope achieved the best averaged accuracy and precision scores for both tested data sets. This is due to the activation frequency of these criteria being much closer to human curation in GenOtoScope than in VIP-HL and InterVar. Notably, VIP-HL and Intervar triggered the commonly implemented 12 criteria much less frequently. To sum up, our choice to implement these 12 criteria, which are refined for HL, can lead to standardized classification results for all HL-relevant genes.

Besides, our implementation of the criteria presents two more advantages: In contrast to the usage of the ANNOVAR annotation tool, licensed for commercial use, we constructed all annotation files needed to examine the ACMG criteria, using freely accessible databases. Thus, we are able to offer GenOtoScope with an open-source software license. Therefore, any interested researcher could update the corresponding code section to produce adjusted annotations to her needs. Equally, the researcher can update the code to change the steps used to examine a given criterion. The second advantage is that GenOtoScope (like VIP-HL) outputs comments for each examined criterion, whereas InterVar does not. This extra information can facilitate the variant curator to justify the activation of a criterion and thus increases the explainability of the automated classification.

### GenOtoScope workflow

**Fig 2:**
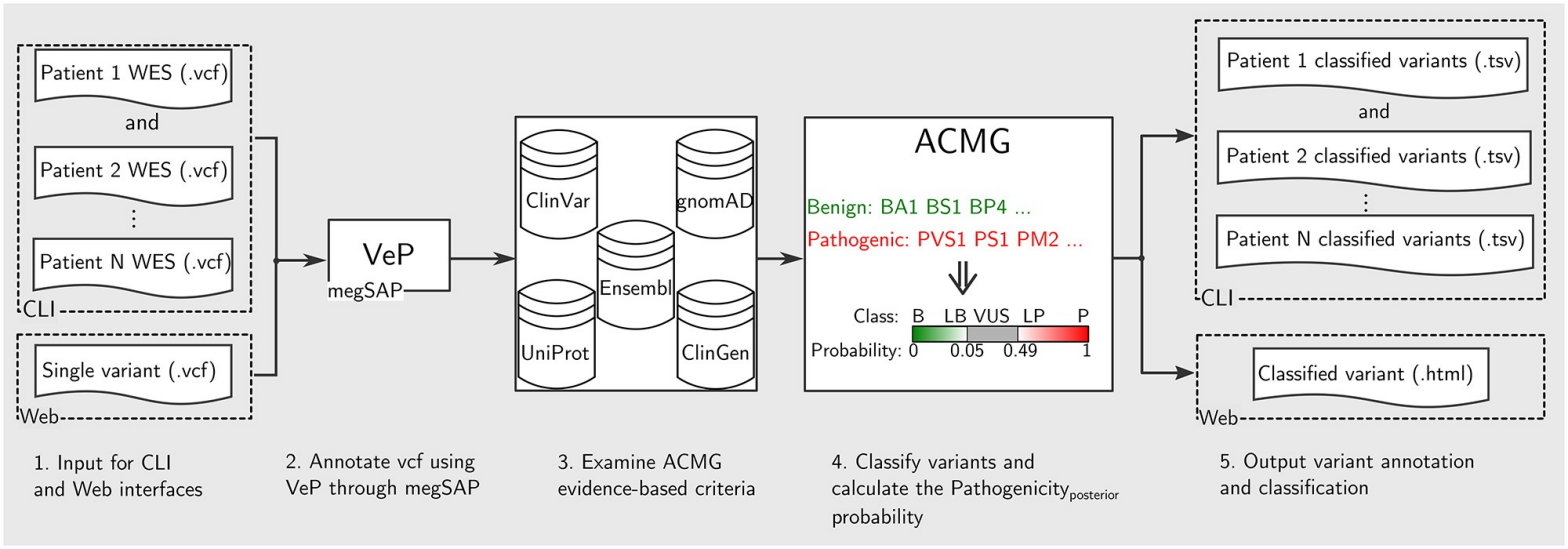
Conceptual workflow of GenOtoScope.

In the following, the methodology to implement the ACMG evidence-based criteria for congenital hearing loss is explained in five key steps. The conceptual workflow of the web and command line interface (CLI) of GenOtoScope is depicted in Fig 2.

In the first step, the user inputs a variant file (.vcf). Depending on the used interface, the input vcf file can contain either a single variant or a larger set of variants of a patient (e.g. full set of WES variants per patient).

Next, functional annotation of the VCF follows, using the VeP annotation tool [16] through the megSAP bioinformatics application (https://github.com/imgag/megSAP). The resulting intermediate variant file (.GSvar) is organized as a standard matrix file (tabular file), where each row is a variant and a column contains variant annotation. These columns contain the basic variant information (for example chromosome position of variant and affected gene name), the transcript and the protein HGVS signature and the unique functional annotation for the variant (e.g. minor allele frequency in gnomAD subpopulations [17], the OMIM variant observed clinical description [18] and the REVEL pathogenicity score for the variant [19]).

The third step uses the core sub-algorithms of GenOtoScope to automatically analyze the listed variants according to ACMG criteria. These sub-algorithms access programmatically four databases: the human clinical variants database ClinVar [20], the human exomes database gnomAD [17], the protein knowledge database UniProt [21] and the clinical genome database [22]. Extracted annotations are organized based on the Ensembl features [23] for a variant-affected transcript. Beyond the mere result of the evaluation of a criterion (activation or non-activation), the tool stores a descriptive comment on the reason of triggering or not of the respective criterion, to be used as an explanation for the user.

In the following step, the tool combines the activated evidence-based criteria to classify the variant into five pathogenicity categories (“benign”, “likely benign”, “VUS”, “probably pathogenic” and “pathogenic”) according to ACMG guidelines. If none of the criteria is activated, the tool classifies the variant as “VUS”. Subsequently, in the same fourth step, GenOtoScope computes the pathogenicity posterior probability based on [12]. This is intended to allow a better discrimination of “VUS” and additional re-classification of “VUS” into benign or pathogenic variants.

In the fifth and final step, GenOtoScope extends the intermediate annotation tabular file with the criteria evaluation results and comments, along with the predicted ACMG class and the computed pathogenicity probability. Finally, the tool saves this file as the produced classification output.

GenOtoScope requires specific annotation files to automate the ACMG criteria based on the previous workflow. These annotations files are: clinical-significant exons, HL-relevant transcripts, critical regions for proteins, critical regions for proteins without benign variants and protein repeat regions without domain intersection. We constructed these annotations files, using publicly available data sets.

### GenOtoScope interfaces

#### Web interface

**Fig 3:**
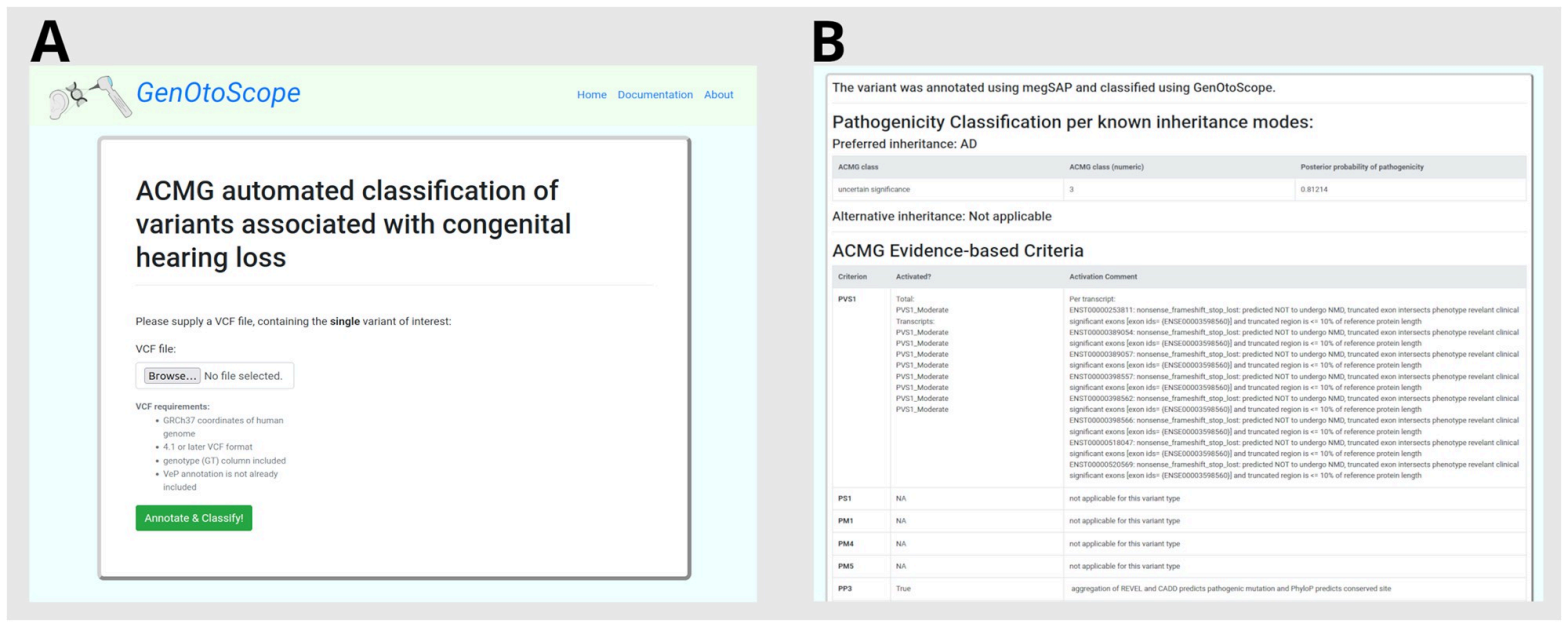
Web interface of GenOtoScope. (A) The home page of the GenOtoScope website. (B) The resulted variant classification page, for an example variant (RS id: 1064797096), which includes its classification based on HL-specified ACMG guidelines.

The web application is targeted for free online usage. Advanced bioinformatics skills are not required. A screenshot of the home page of the GenOtoScope website is shown in Fig 3. Users can upload a single variant file (.vcf) in the home page (Fig 3A). The website will annotate and convert the VCF to GSvar file through the megSAP application. A variant classification page (.html), Fig 3B, will be generated to show the basic annotation of the variant, its ACMG classification and the computed pathogenicity posterior probability.

#### Command line interface

**Fig 4:**
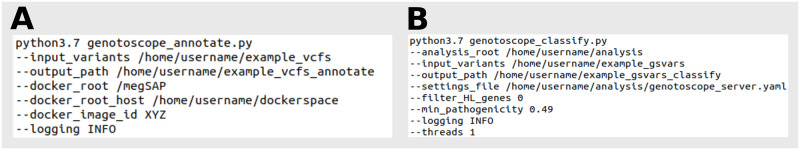
Command line examples for the two commands of GenOtoScope. (A) Annotate all variants presented in VCF files, in input folder, using megSAP application and save results in GSvar files. (B) Classify all variants presented in GSvar files based on ACMG guidelines specified for HL.

The command line interface (CLI) is tailored to bioinformatics personnel. The first command of this mode, genotoscope_annotate.py, accepts as input a folder of VCF files or a single VCF file. It annotates the input VCF files and converts them into GSvar files. The second command, genotoscope_classify.py, accepts as input a folder of GSvar files or a single GSvar file, the output of the previous command. Then, it automatically examines the ACMG evidence-based criteria to classify and computes the pathogenicity posterior probability for each variant in an input GSvar file. The output is an extended GSvar file, containing information on the examination of the ACMG evidence-based criteria, the ACMG pathogenicity class and the pathogenicity posterior probability. Examples of these two commands are shown in Fig 4.

### Automating examination of ACMG evidence-based criteria

In the following subsections, we describe our implementation of the aforementioned 12 ACMG criteria: PVS1 is automated based on [24]. Information from ClinVar database is used for the implementation of PS1 and PM5 (including PM5 Strong). Automation of PM1 examines critical regions provided by [9] and an annotation file containing critical regions without benign mutations, created by a utility script of GenOtoScope. Customized annotation files are also used for (non) repetitive region dependent criteria PM4 and BP3, whereas automation of PP3, BP4 and BP7 employs established prediction algorithms. Population frequency data are taken from gnomAD database, for the implementation of PM2 (PM2 Supporting), BA1 and BS1 (BS1 Supporting). See S3 Appendix, for the software implementation note of GenOtoScope.

#### Refined PVS1

PVS1 criterion is assessed for start-loss, nonsense (stop gained), stop-loss, frameshift, in-frame, splice acceptor and donor variants according to [24].

**Fig 5:**
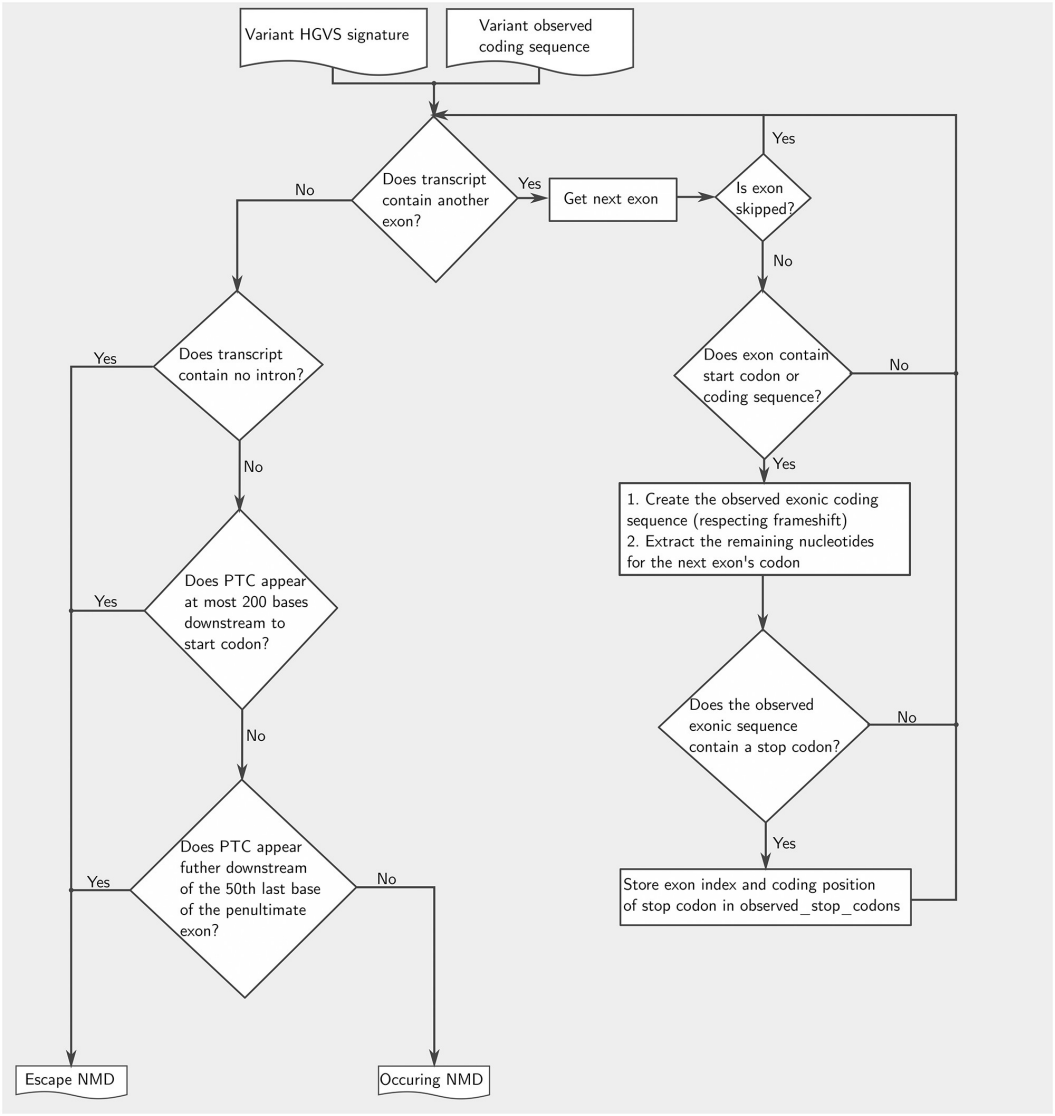
Conceptual flowchart to assess NMD for the refined PVS1 rule.

First, the occurrence of nonsense-mediated decay (NMD) is predicted by a subroutine for each affected transcript using the HGVS signature of the variant to create the observed coding sequence per exon. Altered region is defined as variant-affected coding region. The algorithm locates the 5’-closest stop codon and follows the scheme of [25] to assess the impact of this premature termination codon (PTC) on NMD. The algorithm predicts that the observed coding sequence will escape the NMD, if PTC appears either within the 50 last bases of the penultimate exon, or at most 200 bases downstream from the start codon, or alternatively the transcript contains no introns. Otherwise, NMD is classified to occur. Fig 5 illustrates this subprocess.

If NMD is predicted to occur, the algorithm intersects the stored variant-affected coding region to phenotype-relevant transcripts to decide the PVS1 outcome. If NMD is not predicted to occur, it intersects the variant-affected coding region with protein domain regions. If the result of the intersection is not the empty set, it examines if the affected region overlaps a critical domain for protein function, to decide the PVS1 outcome. If the affected region is not within a known domain, the algorithm examines the overlap of the affected region with the clinically significant exons and the phenotype-relevant transcripts. If such overlap is confirmed, GenOtoScope subprocess investigates whether the PTC results in the removal of more than 10% of the reference protein product.

For start-loss variants, the algorithm first checks if any other transcript contains an alternative start codon. If not, it extracts all potential in-frame start codons which are no further than 200 bases downstream of the lost start codon. Next, it queries the ClinVar database for pathogenic entries with at least one review star between the lost start codon and the detected in-frame start codon. If there is such a ClinVar entry, PVS1 (Moderate) is triggered, otherwise PVS1 (Supporting) is triggered.

See S2 Appendix, sections 1.1.1–1.1.3, for the detailed methodology on the PVS1 annotation files (critical regions for protein function, clinically significant exons, phenotype-relevant transcripts). See S2 Appendix, section 1.2.1 for the results on the PVS1 annotation files.

#### PS1 and PM5 (PM5 Strong)

**Fig 6:**
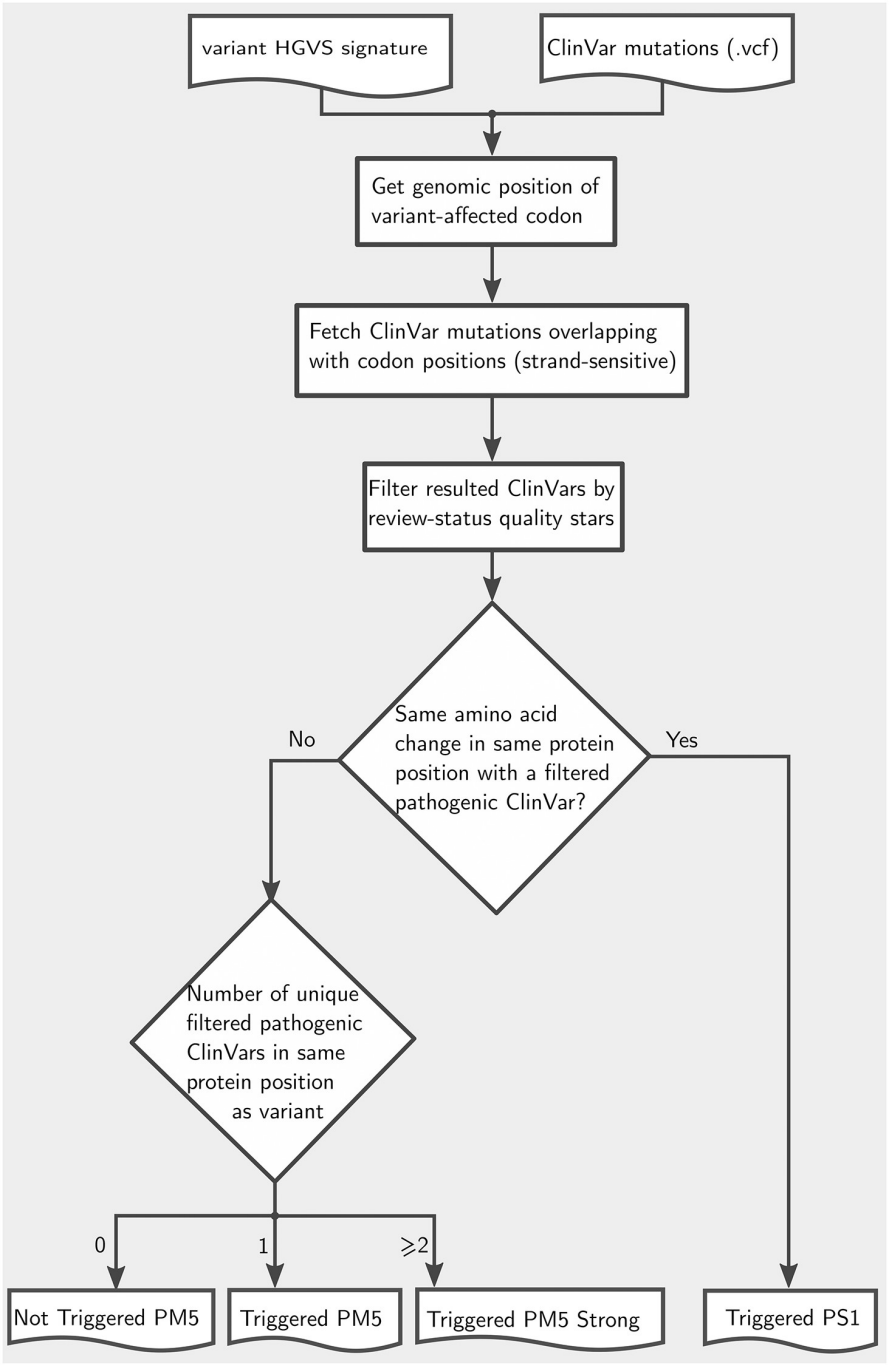
Conceptual flowchart for examining PS1 and PM5 (PM5 Strong).

The workflow of assessing the PS1 and PM5 criteria is shown in Fig 6.

First, genomic positions of the affected codon are computed based on exonic variant location and directionality of the respective gene. Then, all missense variants at corresponding genomic positions are extracted from ClinVar and filtered by strand to match the directionality of the affected gene. Additionally, ClinVar entries can be filtered by a minimum number of review status. This threshold number is user-defined, with default value the minimum of one quality star.

The filtered variants and resulting amino acids are used for further assessment of PS1 and PM5 criteria: PS1 is triggered if any filtered-in variant from ClinVar that is rated as pathogenic results in the same amino acid change as the observed variant. PM5 is triggered if the filtered-in ClinVar entries do not contain the observed amino acid change, but at least one pathogenic variant affecting the same codon. If the entries include two or more such variants, PM5 is applied as strong evidence (PM5 Strong) according to [9].

#### PM1

For automation of PM1 a custom-made annotation file is used. It comprises all critical protein regions without benign ClinVar entries. It also includes the specific domains and motifs of the hearing loss related proteins, defined by [9]. See S1 Appendix, section 1.1, for the detailed implementation of PM1 criterion. In addition, see S2 Appendix, section 1.1.4, for a detailed description of the annotation files used for PM1 implementation.

#### PM4

The algorithm for this criterion is applied to all in-frame (deletions/duplications) and stop-loss variants that do not trigger PVS1 in any strength level. Considering PTC assessed in PVS1 subroutine, length of observed proteins is calculated and compared to the reference protein length. If the variant does not affect a known repetitive region, derived from the UniProt genome annotation, and the resulted protein has a length difference higher than > 10%, PM4 is triggered.

#### BP3

The algorithm for this criterion is applied to all in-frame (deletions/duplications) and nonsense (stop gained) variants. We created an annotation file containing the protein repeat regions not intersecting a protein domain, using the UniProt genome track annotation resource. BM3 is triggered if the variant-affected coding region overlaps a repetitive region without known function.

#### PP3, BP4 and BP7

GenOtoScope incorporates *in silico* tools for conservation (PhyloP [26]), splicing (MaxEntScan [27], dbscSNV [28]) and missense-prediction (REVEL [19], CADD [29]). See S1 Appendix, section 1.2, for details on the aggregation of scores and thresholds.

For missense variants, activation of PP3 requires positive pathogenicity prediction (REVEL/CADD) and high conservation (PhyloP) scores. In contrast, BP4 is triggered if conservation and predicted probability of pathogenicity are low and moreover, the variant is also estimated not to affect splicing (MaxEntScan/dbscSNV).

Variants with no immediate impact on amino acid sequence (exclusion: canonical splice site variants) are similarly screened for potential effects on splicing. If splicing is predicted to be affected and the nucleotide is highly conserved, PP3 is activated. Conversely, if a potential splice variant is predicted to have no splicing effect and conservation is low, BP7 is triggered for synonymous variants and BP4 for other variant types respectively.

#### PM2 (PM2 Supporting) BA1 and BS1 (BS1 Supporting)

Assessment of population data criteria uses user-adjustable minor allele frequency (MAF) thresholds, which by default are the ones defined by [9]. Each gene can be assigned a preferred mode of inheritance, which can be customized by providing an input file. Default settings comprise the inheritance modes of 164 hearing loss gene-disease pairs defined by the VCEP-HL [30]. Also, we included the preferred inheritance patterns for additional genes specified by the HG department of MHH.

For each variant, allele frequencies (AF) of gnomAD subpopulations are retrieved. Known pathogenic variants with high AF are excluded from further assessment of BA1 and BS1 according to [9]. AF of each subpopulation and the median AF of all subpopulations are evaluated with respect to the appropriate inheritance mode threshold. PM2 (PM2 Supporting), BA1 and BS1 (BS1 Supporting) are triggered, if any subpopulation’s AF or the median AF matches the respective inheritance mode threshold.

Regarding different inheritance patterns, the algorithm by default uses distinct thresholds for autosomal dominant and autosomal recessive inheritance mode as specified by [9]. For the X-linked mode of inheritance, autosomal dominant thresholds are adopted. If no mode of inheritance is provided, it is assumed to be unknown. In these cases, the algorithm selects the strictest threshold between autosomal dominant and recessive for each criterion. For mitochondrial genes, the same procedure is used as for unknown mode of inheritance, with an additional warning, since the application of ACMG criteria is validated only for Mendelian disorders.

#### Hearing-loss specific ACMG classification

Having assessed all applicable criteria for a given genomic variant, GenOtoScope combines the activated criteria to compute the respective ACMG class using the five-tier terminology system (“benign”, “likely benign”, “VUS”, “likely pathogenic” and “pathogenic”) defined by [3].

Moreover, GenOtoScope incorporates the extended recommendations of VCEP-HL for the following criteria combinations: (i) Variants triggering PVS1 and PM2 (Supporting) will be classified as “likely pathogenic” for genes associated with autosomal recessive inheritance. (ii) Variants activating BS1 without triggering any pathogenic criterion will be classified as “likely benign”.

#### Computation of pathogenicity probability

After having classified all exome variants of a patient, a number of variants are classified as “VUS”, due to insufficient or conflicting triggered evidence criteria. To help the human curators to discriminate the pathogenicity of the “VUS” cases in a quantitative manner, GenOtoScope calculates the pathogenicity probability for each variant following [12]. The calculation of the pathogenicity probability is calculated automatically for all input variants.

Please see the S1 Appendix, section 1.3, for the parameters values used by GenOtoScope.

## Results and discussion

### Variant classification

#### Data sets

GenOtoScope variant classification was compared to similar tools: (1) InterVar, a tool for variant classification tested across a spectrum of phenotypes [13]; (2) VIP-HL, the recently published tool for hearing loss [11]. We benchmarked the accuracy and precision of variant classification on two data sets.

The first data set is the publicly available set of manually annotated variants by ClinGen VCEP-HL [10], hereafter referred to as VCEP-HL data set. This data set contains manual annotation for 158 variants associated with HL. These variants are contained in 9 HL-relevant genes (*USH2A*, *COCH*, *GJB2*, *KCNQ4*, *MYO7A*, *MYO6*, *TECTA*,*SLC26A4* and *CDH23*). The second data set is the private set of manually annotated variants by the HG department of MHH, hereafter referred to as MHH data set. The MHH data set contains 118 variants, contained in 36 HL-relevant genes. More specifically, the included genes are: *COL11A1*, *USH2A*, *NLRP3*, *OTOF*, *ALMS1*,*PAX3*, *ILDR1*, *WFS1*, *COL11A2*, *COL9A1*, *MYO6*, *SLC26A4*, *CHD7*, *GRHL2*, *TMC1*, *WHRN*, *TNC*, *MYO3A*, *PCDH15*, *CDH23*, *OTOG*, *MYO7A*, *TECTA*, *COL2A1*, *MYO1A*, *P2RX2*, *GJB2*, *GJB6*, *ACTG1*, *MYH14*, *KCNE1*, *TMPRSS3*, *MYH9*, *SOX10*, *POU3F4* and *PRPS1*.

#### Performance metrics

To assess the prediction performance, we combined “benign” and “likely benign” classes to “Benign”, “pathogenic” and “likely pathogenic” classes to “Pathogenic”. Thus, we created a three-class prediction task, containing the “Benign”, “Pathogenic” and “VUS” as the three possible broader classes.

Following the evaluation of the classification tool TAPES [31], we evaluated the accuracy and precision of each software tool, calculating the area under the curve (AUC) of the Receiver Operating Characteristics (ROC) curve and of the precision-recall curve, accordingly.

#### GenOtoScope’s pathogenicity probability refines the classification of “VUS”

We acknowledge that not all evidence-based criteria for HL can be automated, due to the need of further patient’s genomic information and the need for manual curation of certain criteria. Therefore, GenOtoScope currently implements 12 out of the 24 ACMG criteria for HL. Thus, GenOtoScope, which uses the standard classification scheme (S1 Fig), might misclassify a variant as “VUS” even if it belongs in the broader classes of “Benign” or “Pathogenic”.

To investigate GenOtoScope’s classification potential, we provide a refined classification of variants, classified as “VUS”, by original GenOtoScope, based on calculated pathogenicity probability and not the mere classification scheme, following the idea from TAPES classification tool [31]. We will refer to this refined version of GenOtoScope, as GenOtoScope_prob.

Based on [12], the range of values of the pathogenicity probability would be lowered, if a subset of the original ACMG criteria were automatized. Thus, the range of values of the pathogenicity probability calculated for a variant, using the automated criteria by GenOtoScope, will be reduced, compared to the classification provided, by a manual curator, who has evaluated all possible ACMG criteria.

To this end, GenOtoScope_prob reclassifies the “VUS” variants, classified by GenOtoScope, by their calculated pathogenicity probability in the following Alg. 1:

**Algorithm 1** GenOtoScope_prob

**function** REFINE_VARIANTS_OF_UNCERTAIN_SIGNIFICANCE(predicted_class_genotoscope_, pathogenicity_posterior_genotoscope_)

 **if** predicted_class_genotoscope_ = “VUS” **then**

  **if** predicted_posterior_genotoscope_ ≥ 0.49988 **then**

   refined_class ← “Pathogenic”

  **else if** predicted_posterior_genotoscope_ ≤ 0.05072 **then**

   refined_class ← “Benign”

  **else**

   refined_class ← “VUS”

  **end if**

 **else**        ▷ Do not refine “Benign” or “Pathogenic” classifications

  refined_class ← predicted_class_genotoscope_

 **end if**

 **return** refined_class

**end function**

For an immediate comparison with the probability threshold if all criteria were implemented, see S1 Fig.

We have chosen these threshold values, based on relaxing the lowest combination of the triggered criteria needed to predict either one of the broader classes of “Pathogenic” or “Benign” based on [3] and [9]. Consequently, the selection of these thresholds is not dependent on a given test data set, but on the currently implemented ACMG criteria for HL.

Then we have transformed this relaxed combination of criteria to pathogenicity probability based on the pathogenicity probability equation provided in S1 Appendix, section 1.3.

That is, for the “Pathogenic” broader class, the combination of the criteria, with the least pathogenicity strength, resulting in “likely pathogenic” class, is “one pathogenic moderate criterion and at least four pathogenic supporting criteria” ([3] and [9]). However, based on available open data and further patient genetic data, we have implemented seven out of the fourteen ACMG criteria favoring the “Pathogenic” broader class. Therefore, we lowered the combination to “one Moderate and one Supporting criterion” which translates to the probability of 0.49988. Hence, GenOtoScope_prob will refine the “VUS” class, by the original GenOtoScope, to “Pathogenic” for a variant with pathogenicity probability equal to at least 0.49988.

Similarly, for the “Benign” broader class, the combination of criteria with the lowest strength is “at least two benign supporting criteria” to result in the “likely benign” class. GenOtoScope implements five out of the total ten applicable criteria for the “Benign” broader class. Therefore, we reduced the requirements of this combination to be “one benign supporting criterion”, which translates to the pathogenicity probability of 0.05072. Consequently, GenOtoScope_prob will reclassify a variant classified as “VUS”, by GenOtoScope, to the “Benign” broader class if the variant’s probability is lower or equal to 0.05072.

#### Investigation of performance discrepancies

We sought out to investigate the reasons for the discrepancy in prediction performance between the classification tools. To do so, we extended the troubleshooting plots of [32], by calculating the log ratio of the activation frequency of an evidence-based criterion by a classification tool and the manual curation, as:

$$\begin{array}{cc} r_{k}^{e,c} & =log_{10}\left(\frac{\alpha_{k}^{e,c}}{\alpha_{\text{manual}}^{e,c}}\right), \end{array}$$ (1)

where $\alpha_{k}^{e,c}$ is the activation frequency of *e*, any of the implemented ACMG rules, by a tool k={InterVar,VIP-HL,GenOtoScope} for a broader class *c* = {pathogenic, VUS, benign}.

We computed all log ratios for each evidence criterion, *e*, by each classification tool for the three grouped classes, *c*. Finally, we used heatmap plots to depict these log ratios.

### VCEP-HL data set

**Fig 7:**
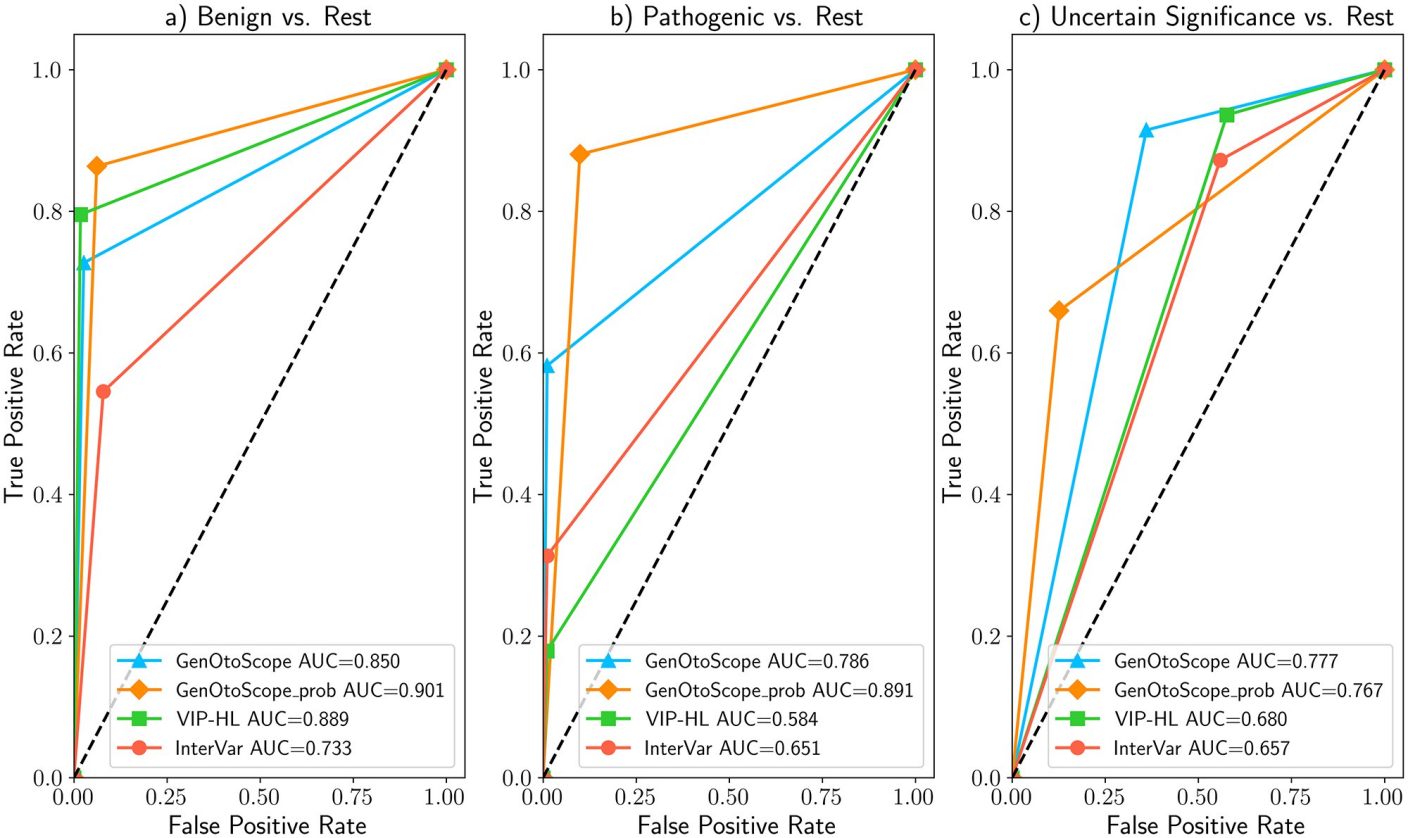
ROC curves and AUC scores of all classification tools for VCEP-HL data set. (A) Prediction of “Benign” broader class versus “Pathogenic” broader class and “VUS” class (B) Prediction of “Pathogenic” broader class versus “Benign” broader class and “VUS” class (C) Prediction “VUS” class versus “Benign” broader class and “Pathogenic” broader class.

**Fig 8:**
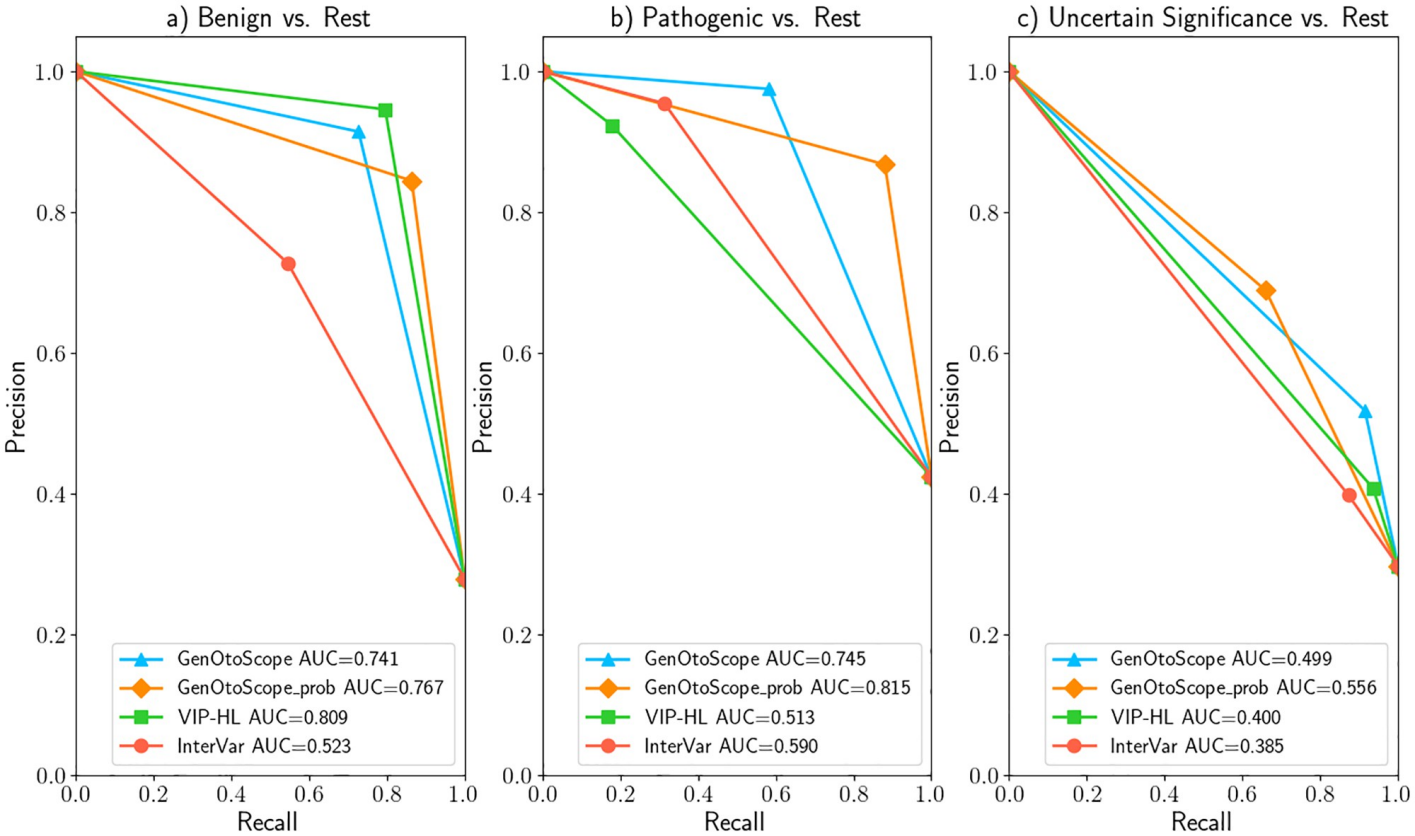
Precision-recall curves and AUC scores of all classification tools for VCEP-HL data set. (A) Prediction of “Benign” broader class versus “Pathogenic” broader class and “VUS” class (B) Prediction of “Pathogenic” broader class versus “Benign” broader class and “VUS” class (C) Prediction of “VUS” class versus “Benign” broader class and “Pathogenic” broader class.

The ROC and precision-recall curves are shown in Figs 7 and 8, respectively. We observed that GenOtoScope and GenOtoScope pathogenicity probability achieved the best AUC scores for all three classes. In Precision-recall curves, VIP-HL achieved slightly higher AUC compared to GenOtoScope for the benign class. However, for the other two classes again GenOtoScope and GenOtoScope pathogenicity probability achieved the best AUC scores. Besides, we calculated the performance scores, AUC of ROC and the average precision of the precision-recall curves for all classification tools. Micro-averaged scores, over the three broader classes (“Benign”, “VUS”, “Pathogenic”), are shown in Table 1. Based on this table, the two versions of GenOtoScope classification achieved the best results for both AUC of ROC and the average precision.

Additionally, we calculated the performance scores, AUC of ROC and the average precision of the precision-recall curves for all classification tools. We show the micro-averaged scores, over the three broader classes (“Benign”, “VUS”, “Pathogenic”) in Table 2. Based on this table, the two versions of GenOtoScope classification achieved the best results for both AUC of ROC and the average precision.

**Table 2:** Micro-averaged performance scores for all classification tools, over the three broader classes in the VCEP-HL data set. Best values of a performance score, across all classification tools, are shown in bold.

Performance scores	Classification Tools			
	GenOtoScope	GenOtoScope_prob	VIP-HL	InterVar
ROC AUC	0.79114	**0.85759**	0.68196	0.65823
Average Precision	0.61342	**0.71960**	0.47307	0.44817

**Fig 9:**
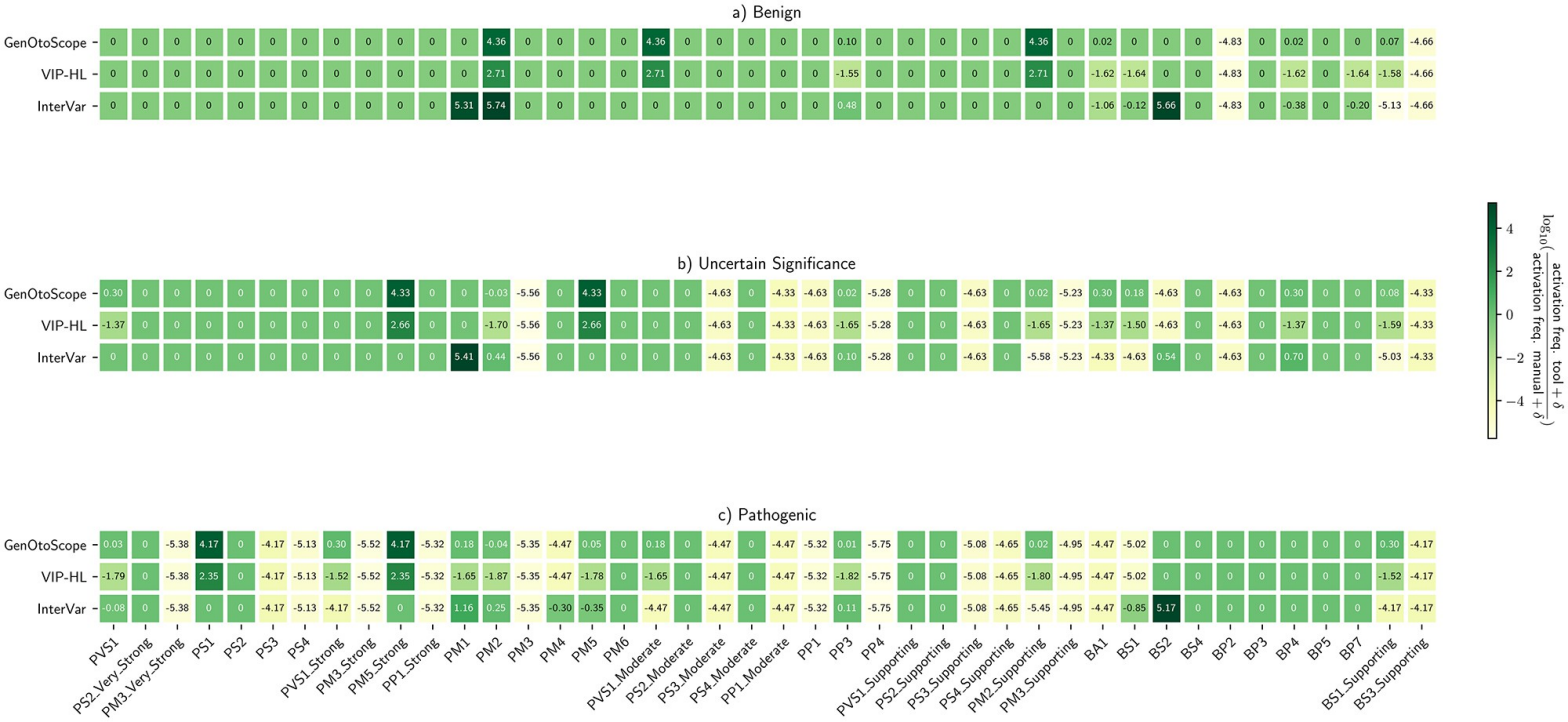
Activation frequency ratios for VCEP-HL data set. Log ratios calculated for each of the three classes classified by the VCEP-HL: (A) “Benign” broader class, (B) “VUS” class and (C) “Pathogenic” broader class.

To explain the difference in prediction performance, we calculated the heatmaps of the log ratio of activation frequency between a classification tool and the manual curation (1). The results are shown in Fig 9.

We observed the following patterns for each grouped class. First, for the “Pathogenic” broader class, VIP-HL activated eight implemented pathogenic rules (PVS1 (Strong), PVS1 (Moderate), PM1, PM5, PVS1 and PM2) from 32 times less (PVS1 (Strong)) to 79 times less (PM2) than the manual curation. Nevertheless, GenOtoScope activated five out of these eight rules with the same frequency as the manual curation (PVS1, PM2, PP3, PM2 (Supporting) and PM5). It activated the remaining three rules (PVS1 (Moderate), PM1 and PVS1 (Strong)) approximately twice as much as the manual curation.

For the “VUS” class, we observed that VIP-HL activated eight implemented rules (BP4, BA1, PVS1, BS1, PM2 (Supporting), PP3 and PM2) from 25 times less (BP4) to 50 times less (PM2) than the manual curation. In contrast, GenOtoScope activated three out of the eight rules (PM2, PP3, PM2 (Supporting)) with the same frequency as the manual curation and it activated the remaining five rules (BS1 (Supporting), BS1, BA1, BP4, PVS1) approximately one to two times more frequently than the manual curation.

For the “Benign” broader class, VIP-HL activated six implemented rules (PP3, BS1 (Supporting), BP7, BP4, BS1 and BA1) from 32 times less (BA1, BS1, BP4, BP7, BS1 (Supporting)) to 40 times less (PP3) than the manual curation. GenOtoScope activated four of these rules (BS1, BP7, BA1, BP4) with approximately the same frequency as the manual curation. The other two rules (PP3 and BS1 (Supporting)) were activated by GenOtoScope, one time more frequently than the manual annotation.

To examine the reasons for the lower precision of GenOtoScope for the “Benign” broader class compared to VIP-HL, despite GenOtoScope’s comparable activation frequency of criteria with manual curation, we examined GenOtoScope misclassifications in this class. That is, we examined the variants belonging in the “VUS” class and misclassified in the “Benign” broader class by GenOtoScope. These misclassified variants are seven, a significant amount for the calculation of precision score, due to the total of 44 variants in the “Benign” broader class. The main reason for the misclassification was that manual annotation used criteria not implemented by GenOtoScope to classify these variants as “VUS”. More specifically, the manual curation used criteria which need manual investigation or not available patient’s family genomic data (for example PP1, PP4 or PM3), to classify five out of these seven variants as “VUS”. The last two variants were misclassified by GenOtoScope pathogenicity probability as their calculated probability was lower than the set threshold for refining a “VUS” as a variant in the “Benign” broader class. VIP-HL could classify correctly three out of these seven variants. Notably, for two out of the three correctly classified variants, the criteria used by VIP-HL, did not correspond to the criteria that should have been activated based on [9].

### MHH data set

**Fig 10:**
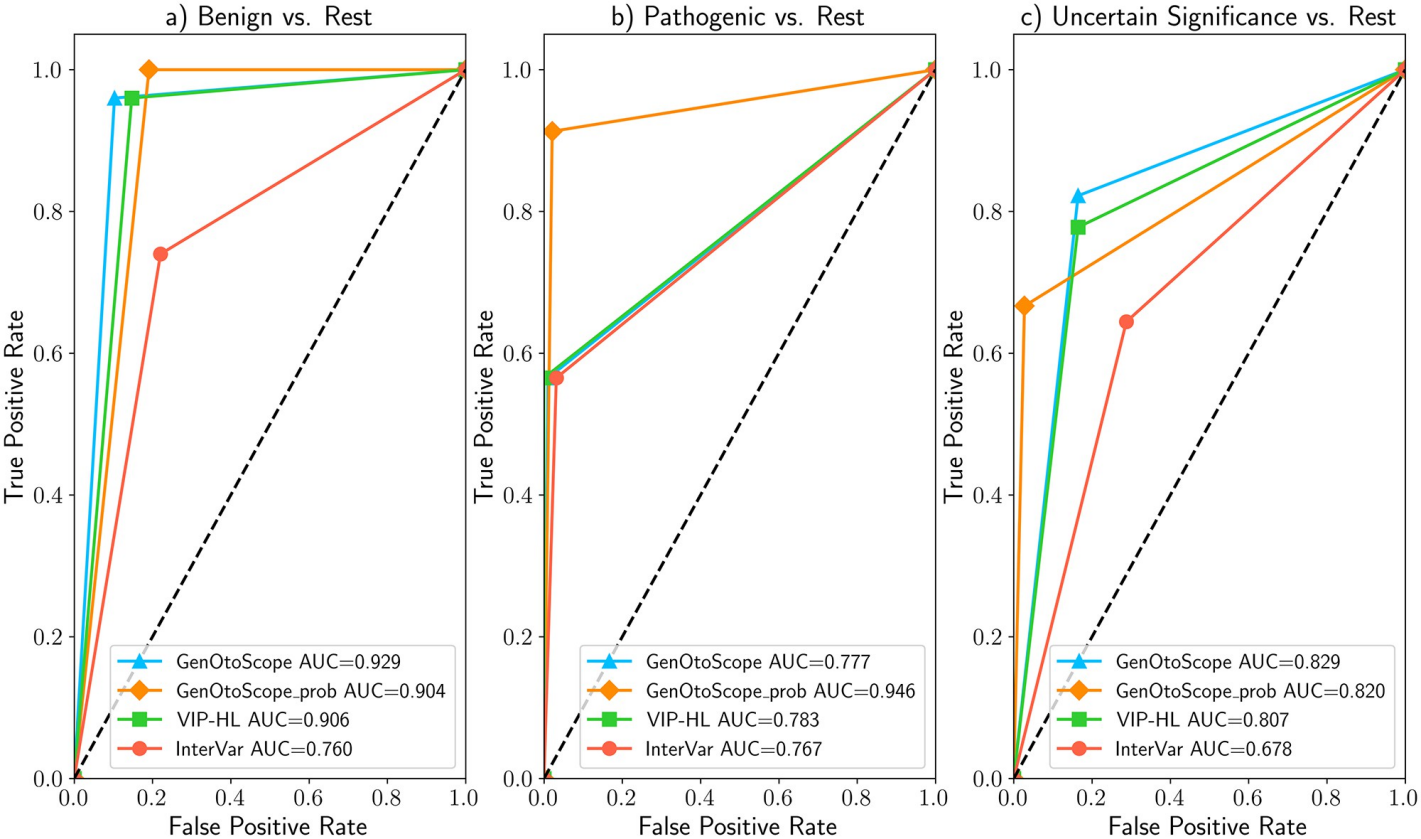
ROC curves and AUC scores of all classification tools for MHH data set. (A) Prediction of “Benign” broader class versus “Pathogenic” broader class and “VUS” class (B) Prediction of “Pathogenic” broader class versus “Benign” broader class and “VUS” class (C) Prediction of “VUS” class versus “Benign” broader class and “Pathogenic” broader class.

**Fig 11:**
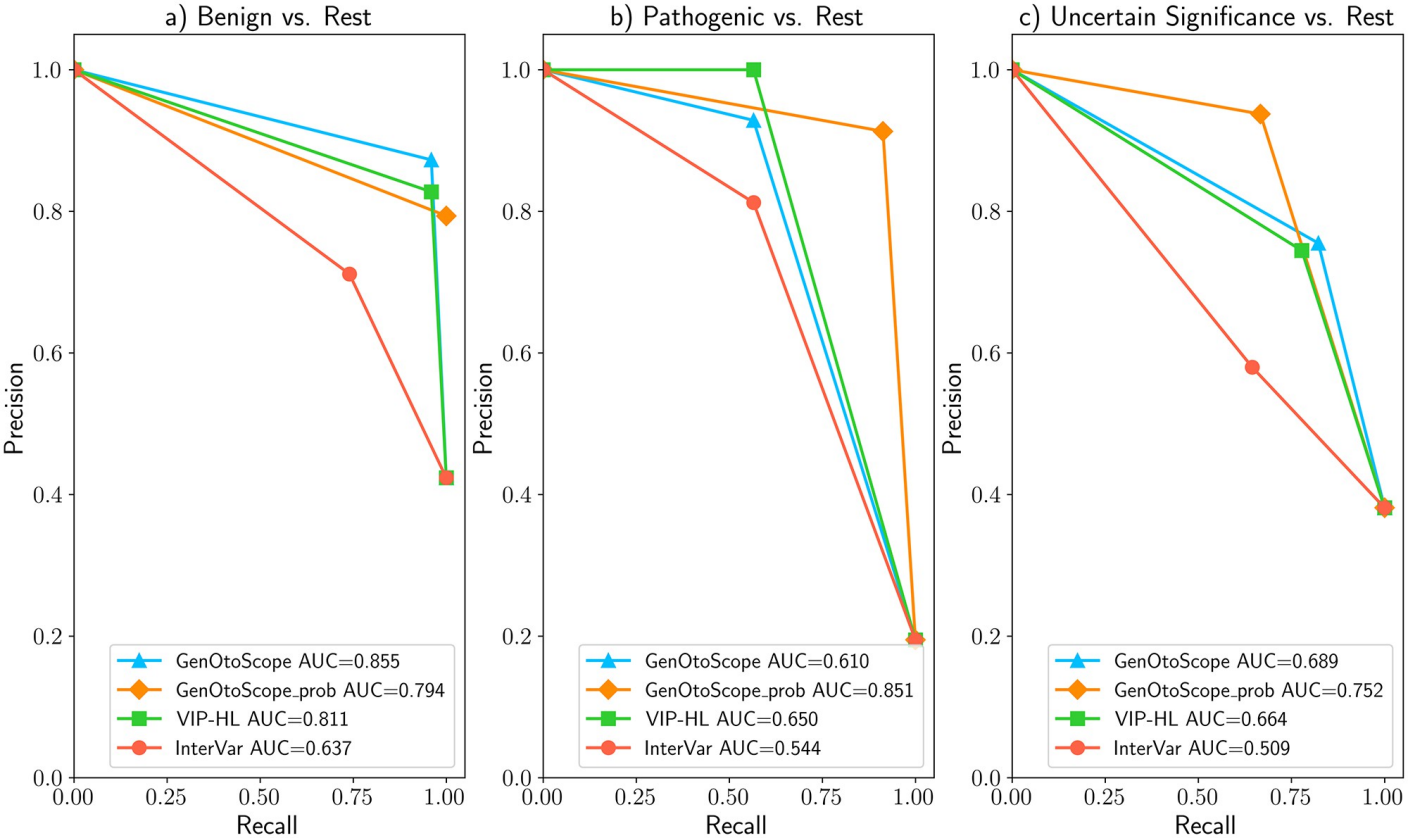
Precision-recall curves and AUC scores of all classification tools for the MHH data set. (A) Prediction of “Benign” broader class versus “Pathogenic” broader class and “VUS” class (B) Prediction of “Pathogenic” broader class versus “Benign” broader class and “VUS” class (C) Prediction of “VUS” class versus “Benign” broader class and “Pathogenic” broader class.

The ROC curve and AUC scores are shown in Figs 10 and 11, respectively. In ROC curves, GenOtoScope or GenOtoScope_prob scored the highest performance values, compared to VIP-HL and InterVar, for all three classes. In the Precision-Recall curves, GenOtoScope outperformed all other classification tools, in terms of AUC score, for benign classification. GenOtoScope and GenOtoScope_prob outperformed all classification tools, in AUC score for pathogenic and “VUS” classes.

We calculated the micro-average AUC of ROC curves and average precision of Precision-Recall curves, across the three broader classes for each classification tool. We show the results in Table 3. As in the previous data set, the two versions of the GenOtoScope classification achieved the best scores for both performance metrics.

**Table 3:** Micro-averaged performance scores for all classification tools, over the three broader classes in the MHH data set. Best values of a performance score, across all classification tools, are shown in bold.

Performance scores	Classification Tools			
	GenOtoScope	GenOtoScope_prob	VIP-HL	InterVar
ROC AUC	0.88701	**0.90395**	0.86441	0.77966
Average Precision	0.73212	**0.76864**	0.68544	0.53085

**Fig 12:**
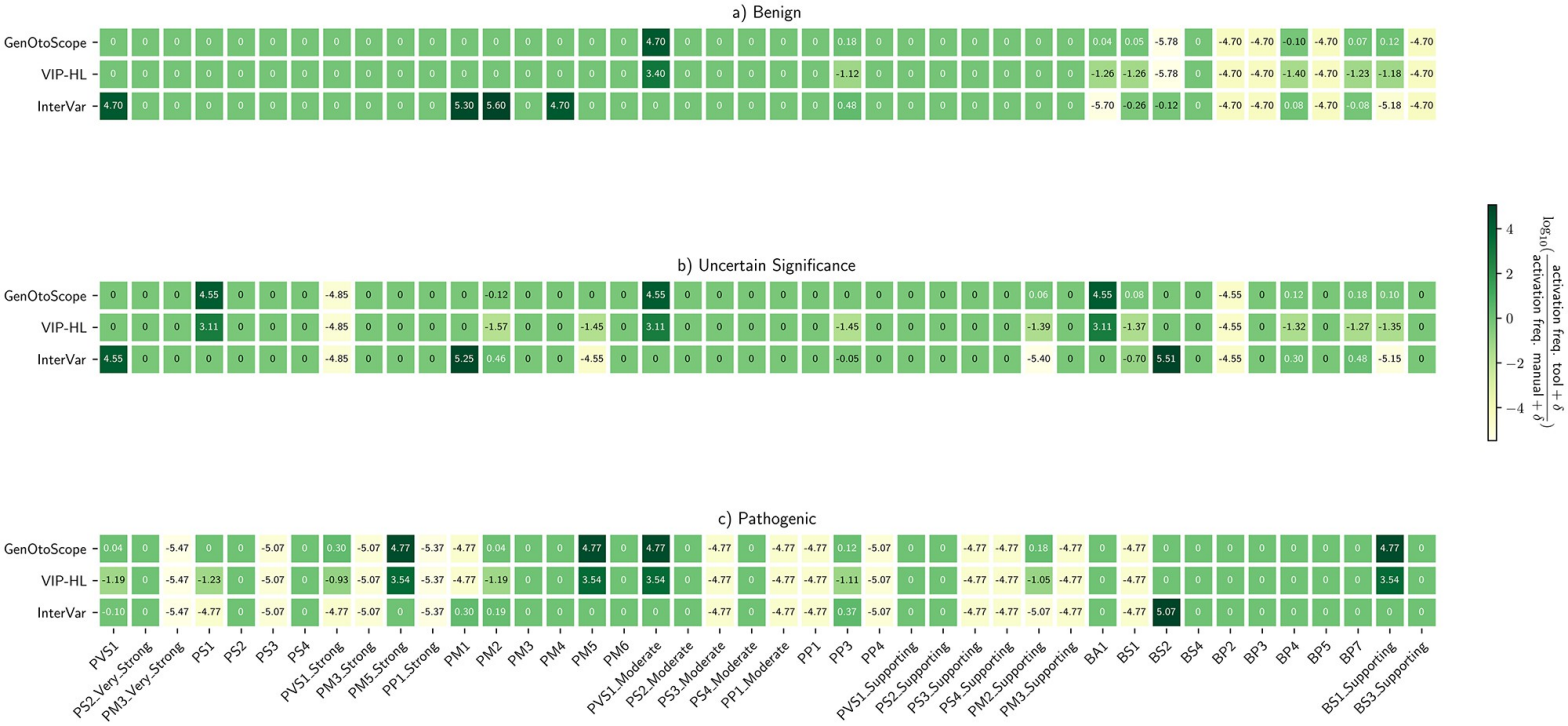
Activation frequency ratios for MHH data set. Log ratios calculated for each of the three classes classified by the MHH manual curators: (A) “Benign” broader class, (B) “VUS” class and (C) “Pathogenic” broader class.

To explain the discrepancy in performance scores, we plotted the heatmaps of log ratio of the activation frequency of a given tool compared to the activation frequency of the manual curation in Fig 12.

For the “Pathogenic” broader class, VIP-HL activated five evidence-based rules (PVS1 (Strong), PP3, PM2, PS1 and PVS1) from eight times less (PVS1 (Strong)) to 16 times less (PVS1). In contrast, for the same class, GenOtoScope activated three out of these eight rules with the same frequency (PVS1, PS1 and PM2) as the manual curation. The remaining two rules were activated approximately one time more (PP3) and twice more often (PVS1 (Strong)) as the manual curation, respectively.

VIP-HL activated eight implemented rules (BP7, BP4, BS1 (Supporting), BS1, PM2 (Supporting), PP3, PM5 and PM2) from 20 times less (BP4 and BP7) to 40 times less (PM2) than the manual curation for the “VUS” class. GenOtoScope activated two of these eight rules (PM5 and PP3) with equal frequency to the manual curation. GenOtoScope activated the remaining six rules (PM2, PM2 (Supporting), BS1, BP4, BS1 (Supporting) and BP7) with approximately one time more (BP7), up to one time less (PM2) as the manual curation.

For the “Benign” broader class, VIP-HL activated six rules (PP3, BS1 (Supporting), BP7, BS1, BA1 and BP4) from 12 times less (PP3) to 25 times less (BP4) than the manual curation. Contrary to VIP-HL pattern, GenOtoScope activated two out of these six rules (BA1 and BS1) with the same frequency as the manual classification and the remaining four rules (BP4, BS1 (Supporting), BP7 and PP3) with approximately two times more (PP3) up to one time less (BP4) as the manual curation.

Based on the observed motives on the activation frequency of each tool compared to the manual curation, we conclude that VIP-HL activated the aforementioned evidence-based rules less frequently than the manual curation. However, GenOtoScope was able to trigger the selected rules with similar or at most twice higher frequency compared to the manual curation. Consequently, we justify the best performance achieved in ROC and Precision-Recall scores by GenOtoScope for all three broader classes compared to the other two classification tools.

## Conclusion

In this work, we present GenOtoScope, an automated classification tool for variants associated with congenital HL. Currently, our tool offers the classification through the automation of 12 out of 24 evidence-based criteria specified for HL [9].

We have shown that GenOtoScope outperformed other variant classification tools in terms of AUC score of ROC curve and of Precision-recall curve for all three broader classes (“Benign”, “VUS” and “Pathogenic”). To explain the difference in performance between the tools, we calculated the ratio of the activation frequency of triggered criteria by each tool and the manual curation. By comparing the ratios for each ACMG criterion, we observed that GenOtoScope achieved the most similar activation frequency to the manual curation, compared to VIP-HL and InterVar.

Besides, the scope of this work is to provide an easily accessible tool to use for the classification of variants for HL phenotype. Therefore, we developed two versions of the tool for two different scenarios. A CLI version to be used by experienced bioinformatics personnel aiming to classify a set of patients WES VCF files and a web interface to be used by other life scientists, with no bioinformatics expertise, to classify a single variant of interest. We hope that this tool will be applied in research settings of molecular genetics to provide a time-efficient and standardized classification of HL variants.

For future extension of GenOtoScope we aim to implement the most frequently activated evidence-based criteria by manual curation to predict the two complementary broader classes. For the “Benign” broader class, the not implemented criteria with highest activation frequency, by the manual curation, were BS2, BP2, BP3, BP5 and BS3 (Supporting). For the “Pathogenic” broader class, the most frequently activated criteria, by the manual curation, were PM3, aggregated for all strengths, PP1, aggregated for all strengths, PS3 and PS4. To implement these criteria which heavily need manual curation, we aim to use databases for genotype to the phenotype such as DisGeNET [33] or to use prediction algorithms to link a mutation of interest to its respective functional study publications, for example AVADA [34]. Last, methods should be implemented to automatically examine the segregation criteria, whenever genomic data of the patient’s family are available. Also, to facilitate even more the personnel with no bioinformatics experience to use the web interface, we would allow the user to input a single variant information, without the need of creating a VCF file on the GenOtoScope website.

Last, by making GenOtoScope an open source project, we aim to facilitate researchers to expand its range of usage to other phenotypes compatible with ACMG-based analysis—for example cardiomyopathy [5] or monogenic diabetes (https://clinicalgenome.org/site/assets/files/7039/clingen_diabetes_acmg_specifications_v1.pdf) – by adjusting specific thresholds, providing customized annotation files and adapting the source code if needed.

## Disclaimer

The classification produced by GenOtoScope is intended for an efficient pathogenicity prediction of WES files, thus for research use only. It is not intended for diagnostic or clinical purposes. The classification provided by GenOtoScope does not replace a physician’s medical judgment and usage is entirely at your own risk. The providers of this resource shall in no event be liable for any direct, indirect, incidental, consequential, or exemplary damages.

## Supporting information

S1 Appendix Detailed description for PM1, PP3, BP4, BP7 criteria implementation and the pathogenicity probability computation. (PDF)

S2 Appendix Constructing annotations for ACMG criteria: Methods & Results. (PDF)

S3 Appendix Software implementation note. (PDF)

S1 Fig ACMG/AMP classification scheme based on evidence-based criteria. The table contains 2 columns. The right column contains sufficient conditions of triggered criteria that result to the left column, pathogenicity class. Sufficient combination of criteria specified for HL are marked with (HL). Pathogenicity probability and its relaxed version are shown for the criteria combinations with the lowest strength that can result to “likely benign” or “likely pathogenic” class.
(TIF)

S2 Fig Conceptual workflow to call critical regions of proteins for assessment of PVS1 rule. (TIF)

S3 Fig Conceptual workflow to call clinical significant exons for PVS1 rule. (TIF)
