# Supplementary material for: GenOtoScope: Towards automating ACMG classification of variants associated with congenital hearing loss
Source: PLoS Comput Biol. 2022 Sep 21;18(9):e1009785. doi: 10.1371/journal.pcbi.1009785 (PMC9529123; doi:10.1371/journal.pcbi.1009785)
Supplement: S1 Appendix — (PDF) [file pcbi.1009785.s001.pdf]

S1 Appendix. Detailed description for PM1, PP3, BP4, BP7 criteria implementation and the pathogenicity probability computation

PM1

The precise regions, used for PM1 criterion, are the pore-forming domain of *KCNQ4* gene and the three-stranded helices of the collagen genes *COL11A2*, *COL4A3*, *COL4A4* and *COL4A5*. PM1 is applied to missense variants overlapping any of the annotated genomic regions. More specifically, if the variant overlaps on the three-stranded motifs of the collagen genes, it accepts only the matches which affect the Glycine residues contained in a Gly-X-Y motif.

PP3, BP4 and BP7

The used thresholds by prediction follow. To decide upon pathogenicity, we aggregate CADD and REVEL in the following scheme: if CADD score is greater than 20, then we set $\mathrm{CADD_{vote}} = 1$, otherwise $\mathrm{CADD_{vote}} = 0$. For REVEL, if REVEL score is greater or equal to 0.7, then $\mathrm{REVEL_{vote}} = 1$, alternatively if REVEL score is lower or equal to 0.15, then $\mathrm{REVEL_{vote}} = 0$, otherwise we set $\mathrm{REVEL_{vote}} = 0.5$. Finally, if the average voting of $\mathrm{CADD_{vote}}$ and $\mathrm{REVEL_{vote}}$ is greater or equal to 1, `GenOtoScope` predicts the variant as pathogenic.

For splicing impact, we aggregate the predictors MaxEntScan and dbscSNV in the following scheme: if $|\frac{\mathrm{observed_{score}} - \mathrm{reference_{score}}}{\mathrm{reference_{score}}}|$ is greater than 0.15, then $\mathrm{MaxEntScan_{vote}} = 1$, otherwise $\mathrm{MaxEntScan_{vote}} = 0$. For dbscSNV, if either ADA score or RF score is greater than 0.6 then $\mathrm{dbscSNV_{vote}} = 1$, otherwise $\mathrm{dbscSNV_{vote}} = 0$. We aggregate the votes similarly to pathogenicity. That is, if the average voting of $\mathrm{MaxEntScan_{vote}}$ and $\mathrm{dbscSNV_{vote}}$ is greater or equal to 1, `GenOtoScope` decides that the variant has a splicing impact.

Last for conservation prediction, we use PhyloP score, as follows: if PhyloP score is greater of 1.6 then `GenOtoScope` decides that this is a conserved site, otherwise that the site is not a conserved site.

Computation of pathogenicity probability

`GenOtoScope` applies the naive Bayes model to calculate the posterior probability of pathogenicity given the triggered ACMG evidence rules using the following equations:

$$\mathrm{Pathogenicity_{posterior}} = \frac{\mathrm{Pathogenicity_{likelihood}} \cdot \mathrm{Pathogenicity_{prior}}}{(\mathrm{Pathogenicity_{likelihood}} - 1) \cdot \mathrm{Pathogenicity_{prior}} + 1} \quad (1)$$

$$\mathrm{Pathogenicity_{likelihood}} = \mathrm{O_{PVST}}^{(\frac{N_{PSU}}{8} + \frac{N_{PM}}{4} + \frac{N_{PST}}{2} + \frac{N_{PVST}}{1} - \frac{N_{BSU}}{8} - \frac{N_{BST}}{2})}, \quad (2)$$

where the default parameters are used: $\mathrm{Pathogenicity_{prior}} = 0.1$, $\mathrm{O_{PVST}} = 350$ and $\mathrm{X} = 2$.

The calculation of the pathogenicity probability is calculated for all input variants automatically.
