# Supplementary material for: GenOtoScope: Towards automating ACMG classification of variants associated with congenital hearing loss
Source: PLoS Comput Biol. 2022 Sep 21;18(9):e1009785. doi: 10.1371/journal.pcbi.1009785 (PMC9529123; doi:10.1371/journal.pcbi.1009785)
Supplement: S2 Appendix — (PDF) [file pcbi.1009785.s002.pdf]

S2 Appendix. Constructing annotations for ACMG criteria: Methods & Results

Methods

In the following, we explain how `GenOtoScope` utility scripts created the needed annotations for the automatic examination of the ACMG criteria. First, we present our methods to construct the annotations for PVS1 criterion: (i) critical regions for protein function, (ii) clinical significant exons and (iii) HL-relevant transcripts. Finally, we present how the respective `GenOtoScope` utility script creates the critical regions for protein function with no benign mutation.

Critical regions for protein function

To automate the assessment of PVS1 rule, `GenOtoScope`'s sub-processes construct three annotation files. The first annotation file includes all critical regions for protein function. To create this file, `GenOtoScope`'s respective sub-process maps all available ClinVar entries to the genomic positions of each UniProt domain, respecting the genomic strand of the domain. Then, it filters-in all mapped ClinVar entries with at least 2 quality stars on their review status field. For each domain, it uses the interpretation field of the filtered-in overlapping ClinVar entries, to calculate the probability of the region being pathogenic (and so critical for protein function) as:

$$\mathrm{P}^{\mathrm{d}}_{\mathrm{pathogenic}} = \frac{N^{d}_{\mathrm{pathogenic}} + \delta}{\sum_{c=\{\mathrm{pathogenic},\mathrm{VUS},\mathrm{benign}\}}(N^{d}_{c} + \delta)}, \qquad (1)$$

where N^{d}_{c} is the number of filtered ClinVar entries, found in the protein domain with UniProt id d, with class equal to c. The parameter $\delta = 10^{-6}$ is used as a smoothing parameter for the probability computation. Finally, the sub-process calls domains with $\mathrm{P}^{\mathrm{d}}_{\mathrm{pathogenic}} \geq 0.51$ as critical for protein function. The sub-process saves all these critical domains for protein function in a BED file containing as columns: their genomic position, strand, protein UniProt id and $\mathrm{P}^{\mathrm{d}}_{\mathrm{pathogenic}}$ probability. The described procedure is depicted in the S2 Fig.

Clinically significant exons

We developed a sub-process to create an annotation file for clinically significant exons, which are exons at which loss of function variants are not frequent in the general population [1]. To do so, the sub-process first aggregates putative loss of function (pLoF) variants of gnomAD [2] per Ensembl exon [3]. Second, for a given exon, it extracts the AF for each subpopulation of a pLoF variant intersecting the exon. Finally, it aggregates the AF of each extracted pLoF variant, for each subpopulation, and if this sum is lower than 0.001 for any subpopulation, the exon is called clinically significant. The output annotation file is in BED format and for each called clinical significant exon, the file contains the columns: genomic position, strand, exon Ensembl id and all containing transcript Ensembl ids. The procedure is depicted in S3 Fig.

Hearing loss-relevant transcripts

The last annotation file for PVS1 contains the hearing loss relevant transcripts. The respective sub-process uses three independent source files to create the hearing loss relevant transcripts and exons. The first file contains the phenotype relevant transcripts and their clinically relevant exons from [4]. The second file contains the disease-gene pairs for hearing loss from ClinGen repository [5]. The third file contains the clinical diagnostics panel for hearing loss created by the HG department of the MHH.

To aggregate these files, we argue that the [4] work contains the most detailed information. This because it contains not only hearing loss relevant transcripts for a given gene, but also the clinically relevant exons of the respective transcripts. In contrast, the two remaining files consist of either disease-gene pairs or only relevant transcripts of genes without specifying their clinically relevant exons. Therefore, the sub-process extends the relevant transcripts and clinically relevant exons reported by [4] with the relevant transcripts and all their contained exons from the diagnostic panel of HG department of MHH. Further, for all genes annotated by ClinGen, but not found in the extended list of annotations of the last intermediate step, it extracts the longest coding transcript or the clinical relevant transcript annotated by Locus Reference Genomic resource (LRG) [6], and their contained transcripts. As final step, it aggregates the ClinGen unique genes annotations [4] with the HG department of the MHH annotations to create the final hearing loss relevant transcripts and clinically relevant exons. The output annotation file is in BED format and for each clinically relevant exon, the file contains the columns: chromosomal position, strand, transcript and exon ids.

Critical regions for protein function with no benign mutation

To automate PM1 evidence-based rule we needed an annotation file containing the critical regions for protein function without benign mutations. To construct these annotated regions, we implemented a similar sub-process as for the critical regions used for PVS1 rule. The only difference is that this sub-process constrains the candidate domains with $P_{pathogenic} \leq 0.51$ (Eq.1) to contain no benign ClinVar mutations. The resulting file is in BED format, containing the same columns as described above for the critical regions for protein function.

Results

PVS1 annotations

`GenOtoScope` sub-processes created the three annotation files needed for the refined PVS1 criterion. For the first file, critical regions, we applied our methodology using 25,552 ClinVar entries, version of March 2021 and 12,776 UniProt domains, version of February 2021. The resulting file contains 1,478 UniProt domains annotated as critical for protein function. Using the HL-relevant transcripts and exons curated in [4], [5] (VCEP-HL) and MHH diagnostic panel, we extracted 2,812 unique exons and 215 unique transcripts, contained in 154 genes. For the annotation file with the clinical significant exons, the used version of the pLoF variants and the allele frequency of exomes of gnomAD was the version 2.1.1. By this process, we annotated 107,966 exons as clinical significant exons.

PM1 annotations

The first annotation file consists of the 10 mutational hotspots relevant to HL as published by VCEP-HL committee, at page[1]. To construct the second annotation file, containing the critical regions for protein function without a benign variant, we followed a modified version of the flowchart used for calling critical regions for protein function (refined PVS1). We used the same versions of ClinVar and UniProt as described above. 750 UniProt domains were called as critical regions, for protein function, containing no benign mutation. The second annotation file provides of these called domain regions. To evaluate the PM1 evidence-criteria, we intersect the chromosomal position of the input variant with both annotation files.

References

1. Abou Tayoun AN, Pesaran T, DiStefano MT, Oza A, Rehm HL, Biesecker LG, et al. Recommendations for interpreting the loss of function PVS1 ACMG/AMP variant criterion. Human mutation. 2018;39(11):1517–1524.

2. Karczewski KJ, Francioli LC, Tiao G, Cummings BB, Alföldi J, Wang Q, et al. The mutational constraint spectrum quantified from variation in 141,456 humans. Nature. 2020;581(7809):434–443.

3. Howe KL, Achuthan P, Allen J, Allen J, Alvarez-Jarreta J, Amode MR, et al. Ensembl 2021. Nucleic acids research. 2021;49(D1):D884–D891.

4. DiStefano MT, Hemphill SE, Cushman BJ, Bowser MJ, Hynes E, Grant AR, et al. Curating clinically relevant transcripts for the interpretation of sequence variants. The Journal of Molecular Diagnostics. 2018;20(6):789–801.

5. DiStefano MT, Hemphill SE, Oza AM, Siegert RK, Grant AR, Hughes MY, et al. ClinGen expert clinical validity curation of 164 hearing loss gene–disease pairs. Genetics in Medicine. 2019;21(10):2239–2247.

6. MacArthur JA, Morales J, Tully RE, Astashyn A, Gil L, Bruford EA, et al. Locus Reference Genomic: reference sequences for the reporting of clinically relevant sequence variants. Nucleic acids research. 2014;42(D1):D873–D878.

[1] `https://submit.ncbi.nlm.nih.gov/ft/byid/vroiax8b/hearing_loss_acmg_specifications_v1_2018.pdf`
