# Supplementary material for: GenOtoScope: Towards automating ACMG classification of variants associated with congenital hearing loss
Source: PLoS Comput Biol. 2022 Sep 21;18(9):e1009785. doi: 10.1371/journal.pcbi.1009785 (PMC9529123; doi:10.1371/journal.pcbi.1009785)
Supplement: S3 Appendix — (PDF) [file pcbi.1009785.s003.pdf]

S3 Appendix. Software implementation note

Implementation note

GenOtoScopeis a software tool written in Python programming language that was developed to automate the examination of the ACMG evidence-based criteria. First, GenOtoScope uses the hgvs library [2] to parse the variant information for each Ensembl transcript. Then, using the HGVS format, the tool constructs the observed coding sequence and it extracts the needed information for the criteria, through the PyEnsembl library[1]. For example, GenOtoScope extracts the start and stop positions of exons and the positions of the start and stop codons. This library uses the Ensembl version 75 for GRCh37 human genome. GenOtoScope uses the PyVCF library[2] to parse variants from VCF files, for instance the ClinVar variants. The tool applies Pybedtools [3] to find the intersections of annotation files, such as the overlap of UniProt domains with repeat regions. Finally, GenOtoScope uses the BioPython library [1] for all other tasks for example, to convert cDNA codons to amino acids. GenOtoScope currently works only for GRCH37 genome assembly coordinates. The performance metrics were calculated using scikit-learn library [4].

The bioinformatics user shall download the whole tool code along with the set of data needed for its execution, e.g. annotation files HL or known variants with high MAF; from the github repository. Besides, at this repository, the user can find example configuration files, example input files (VCF) with the corresponding output files and a documentation on how to install and execute GenOtoScope on a linux machine or server. Last, to be able to use the variant annotation script, genotoscope_annotate.py, the user needs to install the megSAP application on a docker container, as explained on the respective tool github repository [3].

References

1. Cock PJ, Antao T, Chang JT, Chapman BA, Cox CJ, Dalke A, et al. Biopython: freely available Python tools for computational molecular biology and bioinformatics. Bioinformatics. 2009;25(11):1422–1423.

2. Wang M, Callenberg KM, Dalgleish R, Fedtsov A, Fox NK, Freeman PJ, et al. hgvs: a Python package for manipulating sequence variants using HGVS nomenclature: 2018 update. Human mutation. 2018;39(12):1803–1813.

3. Dale RK, Pedersen BS, Quinlan AR. Pybedtools: a flexible Python library for manipulating genomic datasets and annotations. Bioinformatics. 2011;27(24):3423–3424.

4. Pedregosa F, Varoquaux G, Gramfort A, Michel V, Thirion B, Grisel O, et al. Scikit-learn: Machine Learning in Python. Journal of Machine Learning Research. 2011;12:2825–2830.

[1] https://github.com/openvax/pyensembl
[2] https://github.com/jamescasbon/PyVCF
[3] https://github.com/imgag/megSAP
