# Supplementary figures and images for: GenOtoScope: Towards automating ACMG classification of variants associated with congenital hearing loss
Source: PLoS Comput Biol. 2022 Sep 21;18(9):e1009785. doi: 10.1371/journal.pcbi.1009785 (PMC9529123; doi:10.1371/journal.pcbi.1009785)

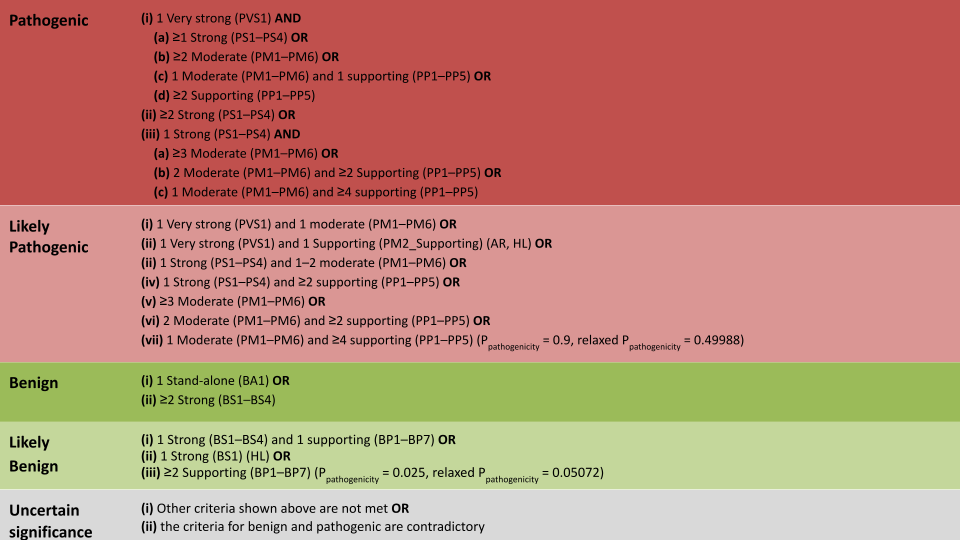

Supplement: S1 Fig — The table contains 2 columns. The right column contains sufficient conditions of triggered criteria that result to the left column, pathogenicity class. Sufficient combination of criteria specified for HL are marked with (HL). Pathogenicity probability and its relaxed version are shown for the criteria combinations with the lowest strength that can result to “likely benign” or “likely pathogenic” class. (TIF) [file pcbi.1009785.s004.tif]

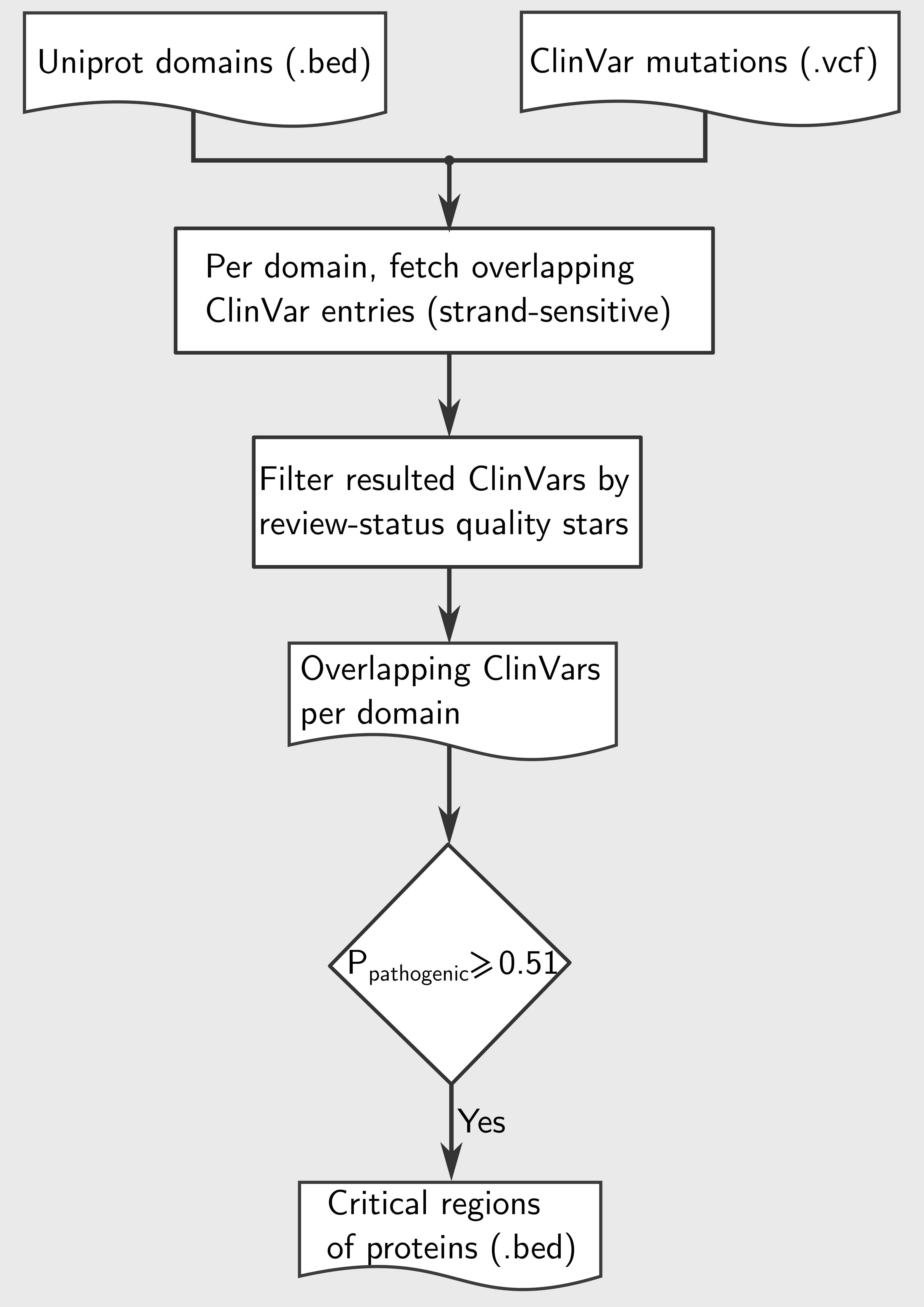

Supplement: S2 Fig — (TIF) [file pcbi.1009785.s005.tif]

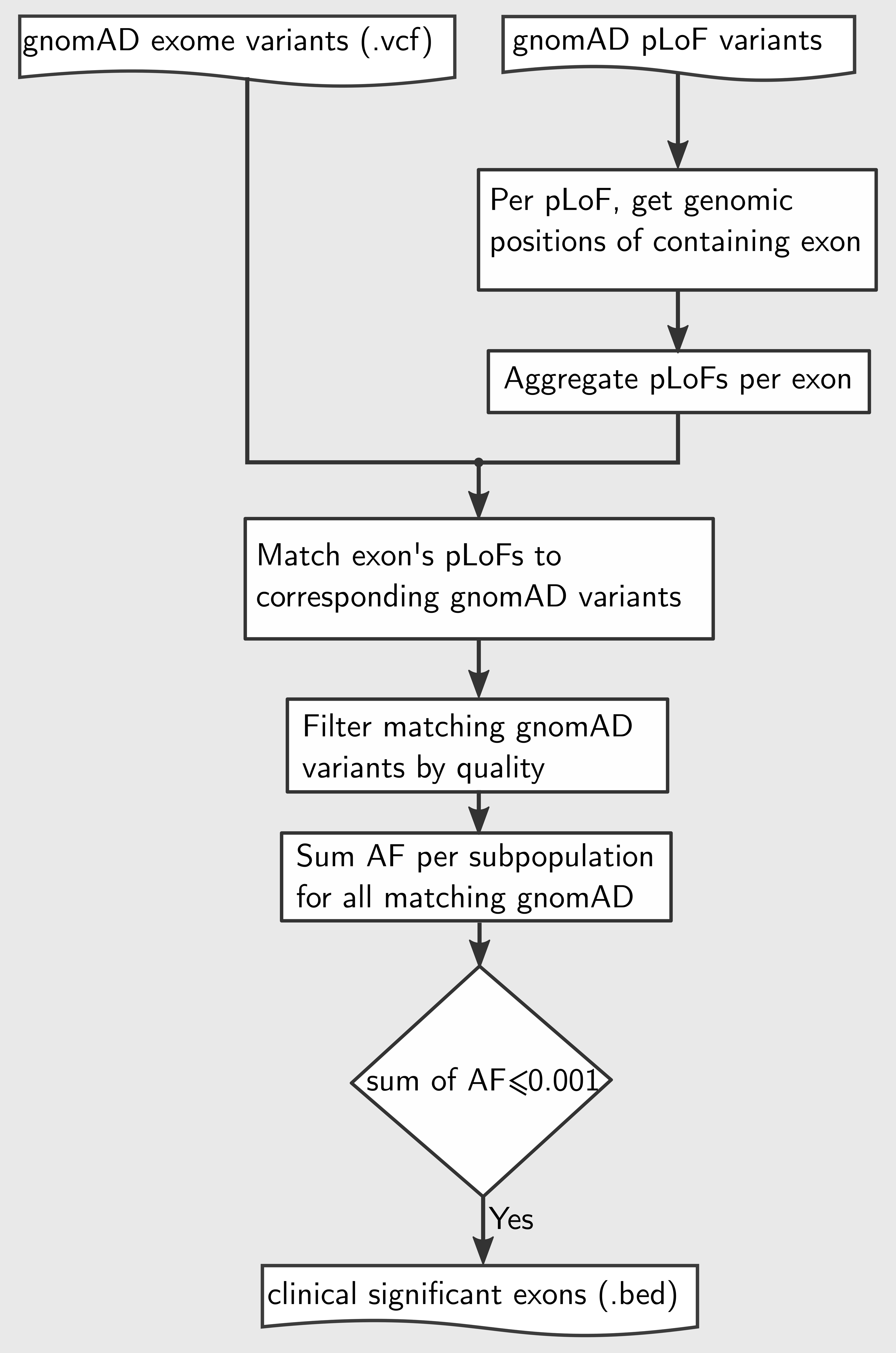

Supplement: S3 Fig — (TIF) [file pcbi.1009785.s006.tif]
